# *Mar*, a MITE family of *hAT* transposons in *Drosophila*

**DOI:** 10.1186/1759-8753-3-13

**Published:** 2012-08-31

**Authors:** Maríndia Deprá, Adriana Ludwig, Vera LS Valente, Elgion LS Loreto

**Affiliations:** 1 Programa de Pós-Graduação em Genética e Biologia Molecular, Departamento de Genética, Universidade Federal do Rio Grande do Sul (UFRGS), Porto Alegre, Rio Grande do Sul, Brazil; 2 Programa de Pós-Graduação em Biologia Animal, Departamento de Zoologia, Universidade Federal do Rio Grande do Sul (UFRGS), Porto Alegre, Rio Grande do Sul, Brazil; 3 Instituto Carlos Chagas (ICC), Fiocruz, Curitiba, Paraná, Brazil; 4 Pontifícia Universidade Católica do Paraná (PUCPR), Curitiba, Paraná, Brazil; 5 Departamento de Biologia, Universidade Federal de Santa Maria (UFSM), Santa Maria, Rio Grande do Sul, 97105-900, Brazil

**Keywords:** MITEs, Buster, hAT, Transposase, *Drosophila*

## Abstract

**Background:**

Miniature inverted-repeat transposable elements (MITEs) are short, nonautonomous DNA elements flanked by subterminal or terminal inverted repeats (TIRs) with no coding capacity. MITEs were originally recognized as important components of plant genomes, where they can attain extremely high copy numbers, and are also found in several animal genomes, including mosquitoes, fish and humans. So far, few MITEs have been described in *Drosophila*.

**Results:**

Herein we describe the distribution and evolution of *Mar*, a MITE family of *hAT* transposons, in Drosophilidae species. *In silico* searches and PCR screening showed that *Mar* distribution is restricted to the *willistoni* subgroup of the *Drosophila* species, and a phylogenetic analysis of *Mar* indicates that this element may have originated prior to the diversification of these species. Most of the *Mar* copies in *D. willistoni* present conserved target site duplications and TIRs, indicating recent mobilization of these sequences. We also identified relic copies of potentially full-length *Mar* transposon in *D. tropicalis* and *D. willistoni*. The phylogenetic relationship among transposases from the putative full-length *Mar* and other *hAT* superfamily elements revealed that *Mar* is placed into the recently determined *Buster* group of *hAT* transposons.

**Conclusion:**

On the basis of the obtained data, we can suggest that the origin of these *Mar* MITEs occurred before the subgroup *willistoni* speciation, which started about 5.7 Mya. The *Mar* relic transposase existence indicates that these MITEs originated by internal deletions and suggests that the full-length transposon was recently functional in *D. willistoni*, promoting *Mar* MITEs mobilization.

## Background

Transposable elements (TEs) are discrete segments of DNA distinguished by their ability to move and replicate within genomes [1]. TE-derived sequences are the most abundant components of several eukaryotic genomes. An increasing amount of evidences shows that TEs can play an important role in driving the evolution and genome complexity [2-6].

TEs can be divided into two classes based on their mechanism of transposition: class I comprises the retrotransposons that transpose through an RNA intermediate, and class II comprises the transposons that transpose through a DNA intermediate [7]. Class II transposons encode for the transposase enzyme, which specifically recognizes the element terminal inverted repeats (TIRs), excises the transposon and inserts it elsewhere in the host genome. Insertion in the genome results in target site duplications (TSDs). Depending on their ability to direct their own transposition, TEs from both classes can include both autonomous and nonautonomous copies. Autonomous TEs encode for the proteins required for their transposition, and nonautonomous TEs can be mobilized *in trans* using the enzymes produced by autonomous elements [7,8].

Within the class II transposons, there is a special group of nonautonomous sequences, called *miniature inverted-repeat transposable elements* (MITEs), which can be present in high number of copies in some genomes. They are characterized by short sequences with no coding capacity, contain conserved TIRs, are flanked by TSDs produced by the insertion and probably originated from a subset of autonomous DNA transposons [9-12]. MITEs often include internal AT-rich sequences that are not homologous to their parental autonomous elements. They were first discovered in plants, but they have also been found in several animal genomes, including *Caenorhabditis elegans*, *Drosophila*, mosquitoes, fish and humans [13,14].

The first MITE families described in *Drosophila* were *Vege* and *Mar*, both of which were discovered in *D. willistoni*[15]. These elements have 884 bp and 610 bp, respectively, and are AT-rich. *Vege* has 12-bp TIRs and *Mar* has 11 bp TIRs, and both elements are flanked by 8-bp TSDs. The initial tBLASTn and BLASTx analysis indicated that both elements have neither coding capacity nor significant sequence similarity to published sequences available at the time that the analysis was conducted. As MITEs have been grouped into TE superfamilies based on the length of their TIRs and TSDs, *Vege* and *Mar* were hypothesized to be members of the *hAT* superfamily [15]. Thus, *Mar* and *Vege* precursor elements are probably autonomous elements from the *hAT* superfamily; however, these precursors were not previously identified. The *hAT* superfamily is widely distributed in multicellular organisms, including plants, animals and fungi [16]. Members of this superfamily are flanked by 8-bp TSDs, have relatively short TIRs (5 to 27 bp) and are less than 4 kb in overall length [7]. Recently, the *hAT* superfamily was divided into two families, *Ac* and *Buster*, primarily due to differences in target site selection [17].

Little is known about MITEs in *Drosophila*.We investigated the presence and evolution of *Mar* in Drosophilidae species and characterized *Mar* copies from the *D. willistoni* genome. We show herein that *Mar* is restricted to the *willistoni* subgroup species and propose that *Mar* originated prior to the diversification of these species. In *D. willistoni*, we found evidence of recent mobilization and amplification. We also identified relic copies of a full-length *Mar* in *D. tropicalis* and *D. willistoni*, suggesting that the origin of the *Mar* MITEs occurred by internal deletion of an autonomous copy followed by amplification. In a phylogeny of *hAT* elements, full-length *Mar* forms a clade with *Buster* elements from bat, mosquito, sea urchin (*Strongylocentrotus purpuratus*), zebrafish (*Danio rerio*) and freshwater planarian (*Schmidtea mediterranea*), and not with other *Drosophila hAT* elements. The TSD consensus also indicates that *Mar* is a *hAT* element from the *Buster* family. As far as we know, this is the first *Buster* element described in *Drosophila*.

## Results

### *Mar* is restricted to the *willistoni* subgroup species

*In silico* searches for *Mar* homologous sequences were conducted in the following genomes: *D. melanogaster*, *D. simulans*, *D. sechellia*, *D. yakuba*, *D. erecta*, *D. ficusphila*, *D. eugracilis*, *D. biarmipes*, *D. takahashii*, *D. elegans*, *D. rhopaloa*, *D. kikkawai*, *D. ananassae*, *D. bipectinata*, *D. pseudoobscura*, *D. persimilis*, *D. willistoni*, *D. mojavensis*, *D. virilis* and *D. grimshawi*. As expected, sequences homologous to *Mar* were found in *D. willistoni*. In the other 19 available genomes, no sequences homologous to *Mar* were found. These available genomes comprise three species from the *Drosophila* subgenus and 16 from the *Sophophora* subgenus, including 14 species from the *melanogaster* group and 2 from the *obscura* group.

**Figure 1:**
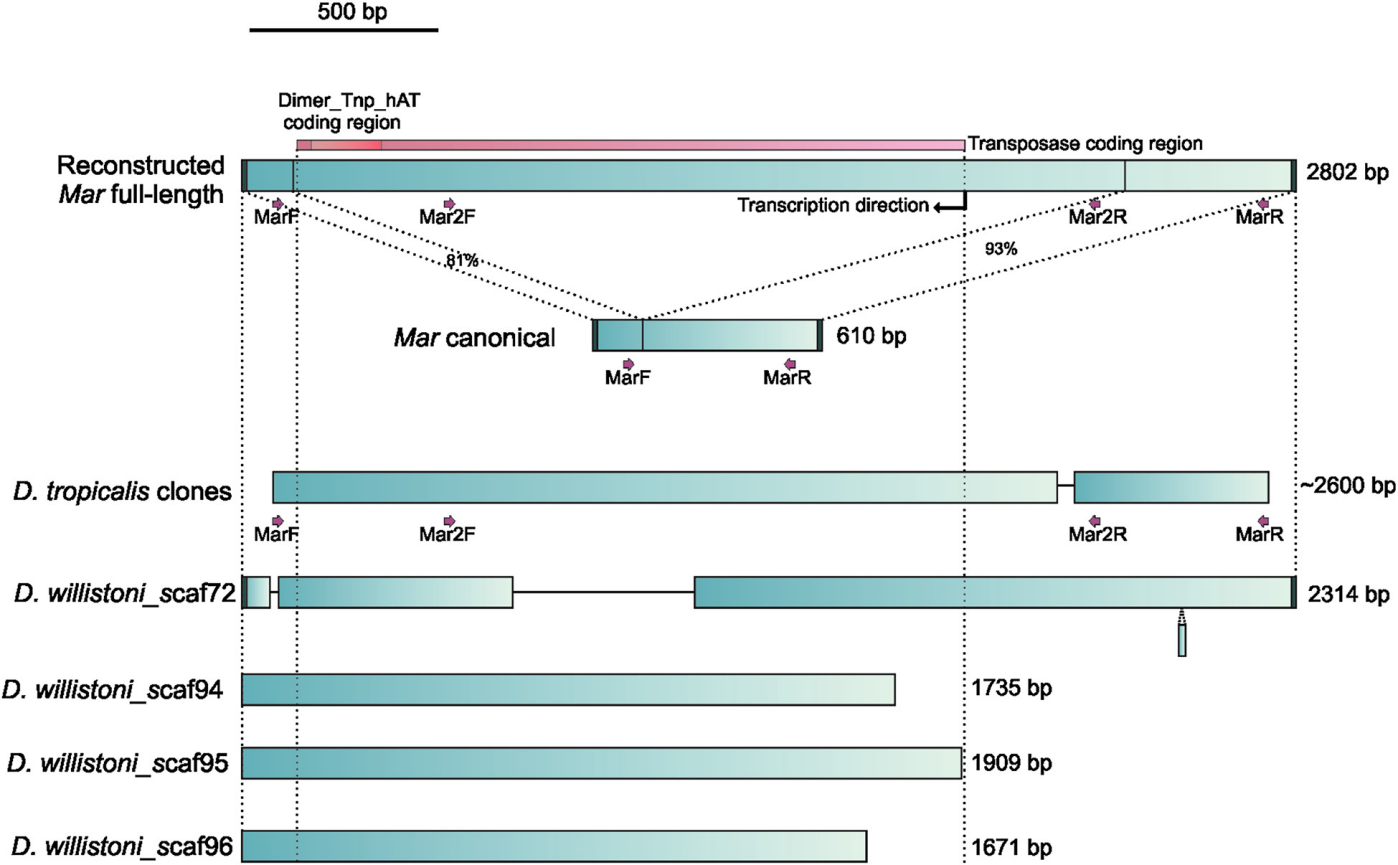
**Schematic representation of the reconstructed full-length *Mar *compared to the canonical *Mar *element (MITE).** Common regions are indicated, including terminal inverted repeats (black boxes). The transposase coding region with the Dimer_Tnp_hAT domain coding region is also shown. Below are the schematic representations of copies found in *D. tropicalis* and *D. willistoni*. Only indels of more than 12 nucleotides are represented. Arrows indicate the primer annealing regions. The primers MarF and MarR were used to amplify *Mar* from the *willistoni* group species, and Mar2F and Mar2R were used to sequence the *D. tropicalis* clones

**Figure 2:**
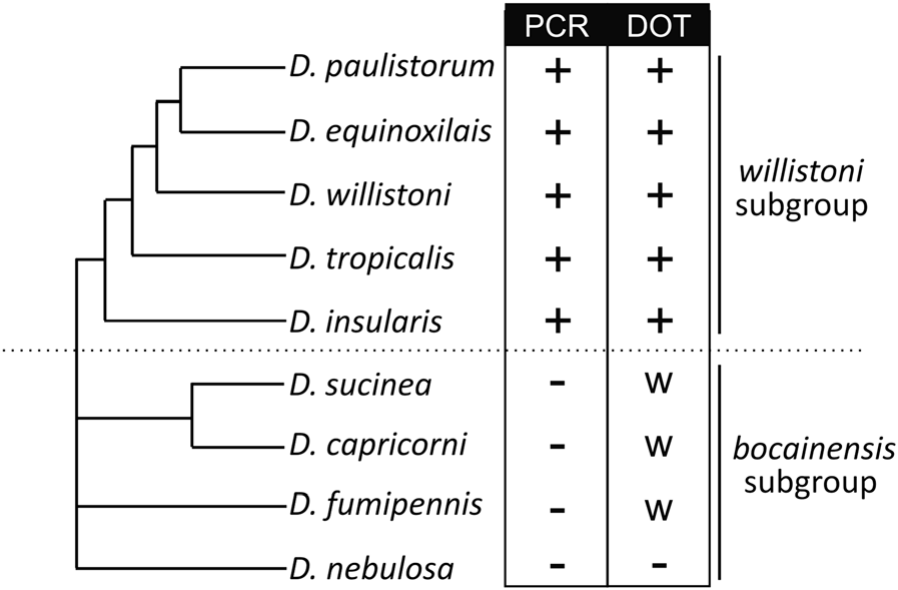
**Evolutionary relationships between the*willistoni*group species, based on**[25]**, and the results obtained from the PCR and Dot blot screenings.**

To expand the analysis of *Mar* distribution, we used PCR and Dot blot strategies in a large number of Drosophilidae species belonging to different *Drosophila* groups (Table 1). A pair of primers, MarF and MarR, was used to amplify a 455-bp fragment of *Mar* (Figure 1). PCR results showed amplification only in the species from the *willistoni* subgroup: *D. willistoni*, *D. paulistorum*, *D. equinoxialis*, *D. insularis* and *D. tropicalis.* The fragment lengths varied from roughly 270 bp to 450 bp for most species, but for *D. tropicalis *the amplified fragment was larger than expected (approximately 2,600 bp), suggesting the possibility of finding a full-length transposon. The Dot blot results (Additional file 1 and Figure 2) corroborated the PCR results, showing positive signals only in the *willistoni* subgroup species. Species from the *bocainensis* subgroup (also part of the *willistoni* group) presented a very weak signal, which may indicate the presence of highly divergent sequences related to *Mar*.

**Table 1:** The Drosophilidae species investigated in this work, their taxonomic placement and their respective PCR and Dot blot results

**Genus**	**Subgenus**	**Group**	**Species**	**PCR**	**Dot blot**
*Drosophila*	*Drosophila*	*guarani*	*D. ornatifrons*	-	-
			*D. subbadia*	-	-
			*D. guaru*	-	-
		*guaramuru*	*D. griseolineata*	-	-
			*D. maculifrons*	-	-
		*tripunctata*	*D. nappae*	-	-
			*D. paraguayensis*	-	?
			*D. crocina*	-	-
			*D. paramediostriata*	-	-
			*D. tripunctata*	-	-
			*D. mediodifusa*	-	?
			*D. mediopictoides*	-	-
		*cardini*	*D. cardinoides*	-	?
			*D. neocardini*	-	-
			*D. polymorpha*	-	-
			*D. procardinoides*	-	?
			*D. arawakana*	-	?
		*pallidipennis*	*D. pallidipennis*	-	?
		*calloptera*	*D. ornatipennis*	-	-
		*immigrans*	*D. immigrans*	-	-
		*funebris*	*D. funebris*	-	-
		*mesophragmatica*	*D. gasici*	-	-
			*D. brncici*	-	?
			*D. gaucha*	-	-
			*D. pavani*	-	?
		*repleta*	*D. hydei*	-	-
			*D. mercatorum*	-	-
			*D. mojavensis*	-	-
			*D. buzzati*	-	?
		*canalinea*	*D. canalinea*	-	-
		*flavopilosa*	*D. cestri*	-	?
			*D. incompta*	-	-
		*virilis*	*D. virilis*	-	-
		*robusta*	*D. robusta*	-	-
	*Sophophora*	*melanogaster*	*D. melanogaster*	-	-
			*D. simulans*	-	-
			*D. sechellia*	-	?
			*D. mauritiana*	-	-
			*D. teissieri*	-	-
			*D. santomea*	-	-
			*D. erecta*	-	-
			*D. yakuba*	-	-
			*D. kikkawai*	-	-
			*D. ananassae*	-	-
			*D. malerkotliana*	-	-
			*D. orena*	-	-
		*obscura*	*D. pseudoobscura*	-	-
		*saltans*	*D. prosaltans*	-	-
			*D. saltans*	-	-
			*D. neoelliptica*	-	-
			*D. sturtevanti*	-	-
		*willistoni*	*D. sucinea*	-	W
			*D. nebulosa*	-	-
			*D. capricorni*	-	W
			*D. fumipennis*	-	W
			D. willistoni^*^	+	+
			D. paulistorum^*^	+	+
			*D. insularis*	+	+
			*D. tropicalis*	+	+
			*D. equinoxialis*	+	+
	*Dorsilopha*		*D. busckii*	-	-
*Zaprionus*			*Z. indianus*	-	-
			*Z. tuberculatus*	-	-
*Scaptodrosophila*			*S. latifasciaeformis*	-	-
			*S. lebanonensis*	-	-

*More than one strain was used for these species. *D. willistoni* strains: ww, 17A2 and WIP4. *D. paulistorum* strains: Ori (semispeciesOrinocan), Andi and PR (semispecies Andean-Brazilian). (-) No amplification or hybridization signal; (+) positive amplification or hybridization; w, weak hybridization signal; ?, not tested.

**Figure 3:**
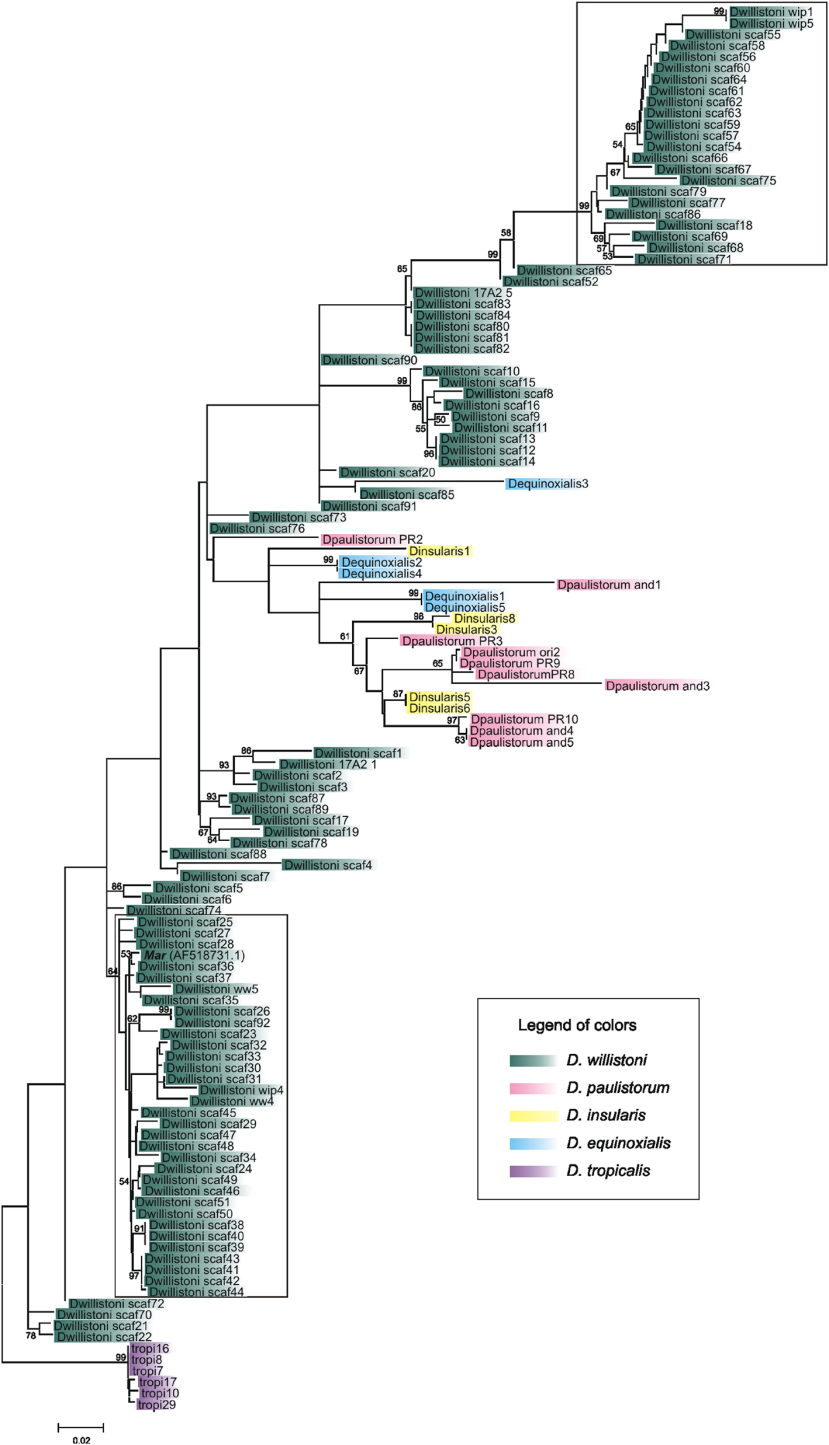
***Mar*Neighbor-joining tree.** Bootstrap values are shown at the nodes; values smaller than 50 were omitted. Different species are highlighted with different colors, as shown in the legend. The two clades highlighted with rectangles represent two events of pronounced copy number amplification in *D. willistoni*. More information about these sequences is available in Additional files 2 and 3.

All cloned sequences (five from *D. insularis*, ten from *D. paulistorum*, five from *D. equinoxialis*, seven from *D. willistoni* and six from *D. tropicalis*) and those obtained by *in silico* searches (93 sequences from the *D. willistoni* genome) were used in the phylogenetic analysis to understand the evolutionary dynamics of *Mar* in the *willistoni* subgroup (GenBank accession number and scaffold coordinates of sequences are shown in Additional files 2 and 3). Figure 3 shows the Neighbor-joining tree obtained for *Mar*, which can be compared with the host species phylogeny in Figure 2. Two major groups, highlighted in the phylogeny, are composed of only very similar sequences from *D. willistoni*. Most of the sequences from *D. equinoxialis*, *D. paulistorum* and *D. insularis* are located in a group with very low branching support. The maximum likelihood (ML) and Bayesian trees show similar topologies (data not shown).

The *Mar* sequences present an overall mean divergence of 9.96%. Table 2 shows the mean divergence of *Mar* sequences found within and between species. The intraspecies divergence ranged from 0.3% for *D. tropicalis* up to 8.6% for *D. paulistorum*. Concerning the interspecies divergence, the values varied from 8.5% between *D. paulistorum* and *D. insularis* to 16.3% between *D. tropicalis* and *D. paulistorum*. Lower levels of intraspecies divergence would be expected if the copies were recently transposed. The generally high divergence found within species and the interspersed distribution of species in the phylogeny can be explained by the presence of these sequences prior to the split of the species. On the other hand, in *D. willistoni*, we were able to evaluate a large number of copies, which enabled us to obtain a better view of *Mar* evolution. In spite of the presence of ancient *Mar* copies in *D. willistoni*, represented by their distinct positions in the phylogeny, there are two clear events of recent mobilization and pronounced amplification of *Mar* (highlighted clades in Figure 3).

**Table 2:** **Nucleotide divergence percentages of*Mar*sequences found within and between species**

**Species**	***D. paulistorum***	***D. insularis***	***D. equinoxialis***	***D. willistoni***	***D. tropicalis***
*D. paulistorum*	8.6				
*D. insularis*	8.5	7.0			
*D. equinoxialis*	11.2	9.9	7.4		
*D. willistoni*	14.1	12.7	11.5	8.2	
*D. tropicalis*	16.3	14.1	13.3	14	0.3

### *Mar* copies from *D. willistoni*

We identified 93 *Mar* sequences in the *D. willistoni* genome (Additional file 3). The exact number of copies is difficult to determine because the genome contains some small and fragmented copies that are not captured in the searches. Also, we cannot exclude the existence of duplicated scaffolds in the database, particularly the very short ones. Of the sequences identified, 74 (79%) contain 11-bp conserved TIRs (CAG(G/A)GGTAGGC), which are not perfect as they were described previously [15]. Only one sequence exhibited perfect TIRs. The majority of copies (79%) are flanked by 8-bp conserved TSDs, indicating recent mobilization of these sequences. The *Mar* element TSD consensus sequence (5^′^-nnnTAnnn-3^′^) matches that of the *Buster* element TSD consensus sequence. This strongly suggests that *Mar* belongs to the *Buster* family of *hAT* transposons. Analysis of *Mar* copies distribution throughout the genome reveals that 32 copies are found within a gene or less than 2 kb from a gene (Additional file 4). Only a small region of *Mar* was found in a predicted coding sequence.

### Putative full-length *Mar*

The amplified sequences from *D. tropicalis* were much longer than expected. We therefore used a second pair of primers, Mar2F and Mar2R, to sequence the entire fragment. We obtained six clones with good-quality sequences of approximately 2,480 bp with a 300-bp region homologous to *Mar* in the 3^′^ and few nucleotides in the 5^′^ region. These clones have 96% to 99% sequence identity and show a mean divergence of 10.5% from the corresponding region of the canonical *Mar* sequence. We cannot distinguish whether these clones are different copies or alleles from the same genome, or if there is polymorphism in the population. BLASTn analysis of these sequences showed significant sequence similarity to the *Mar* element and did not produce any other significant hit.

For all clones, the FGENESH program failed to identify a significant coding region. However, BLASTx searches revealed an intriguing similarity to proteins belonging to the TFII-I family in several distinct organisms, including *Camponotus floridanus* (insect), *S. purpuratus* (sea urchin), *Anoliscaro linensis* (lizard) and several fishes. The highest similarity corresponded to the general transcription factor II-I repeat domain-containing protein 2-like from *Xenopus tropicalis* (XP_002941054), and the BLAST alignments showed significant similarity (query coverage: 70%; E-value: equal to or less than 5e-92; mean similarity: 50%), except for the presence of stop codons in the *D. tropicalis* sequences. The *D. tropicalis* sequences also showed similarity to some transposase sequences, although they had lower similarity scores, confirming their TE origin. This *X. tropicalis* protein could be an element that was incorrectly annotated, since a CENSOR screening against a Repbase reference collection of repeats revealed 66% similarity with hAT-43_SM, an element from *S. mediterranea*. Alternatively, this protein could have resulted from the domestication of a *hAT* superfamily element that has not yet been described. There are several examples of elements from this superfamily being exapted to essential functions within the host genome [17,18].

To better characterize the sequences found in *D. tropicalis*, we searched for similar sequences in the *D. willistoni* genome and found four sequences with significant similarity (mean of 88%). A schematic representation of these sequences can be observed in Figure 1, and the scaffold coordinates are available in Additional file 3. Three sequences (scaf94, scaf95 and scaf96) are shorter than those from *D. tropicalis*, but the other one (scaf72) drew our attention because its TIR sequences are identical to those found in the canonical *Mar* element and it is flanked by 8-bp TSDs with one mismatch (CTCTAC(C/T)C). Despite the fact that this appears to be a complete element, we were not able to find a significant coding region in this copy or in the shorter copies.

Next we aligned the canonical *Mar*, the *D. tropicalis* sequences and the Dwillistoni_scaf72 sequence from *D. willistoni* to obtain a consensus sequence by selecting the most common nucleotide in each position. Some slight modifications were made to the consensus sequence in an attempt to reconstruct a functional sequence with potential coding regions. An alignment of these sequences can be found in Additional file 5. Using this approach and the FGENESH program, we were able to identify a well-defined exon predicted to encode a protein of 591 amino acids. As expected, a BLASTp search also showed significant similarity to the *X. tropicalis* protein (XP_002941054), and a *hAT* family dimerization domain was found in the carboxy terminal of the predicted protein (Additional file 6). A schematic representation of this reconstructed *Mar* full-length element and the transposase coding region is shown in Figure 1. The sequences of the entire reconstructed element, coding region and protein are available in Additional file 7.

Although the *D. willistoni* complete copy (scaf72) has a large deletion in relation to the reconstructed copy and has no coding capacity, the presence of TIRs identical to those present in the canonical *Mar* element indicates that this is a relic of an autonomous full-length *Mar*. Not surprisingly, this sequence appears as a basal branch in the *Mar* phylogeny (Figure 3). Moreover, the TSD sequences flanking this copy also match the *Buster* element TSD consensus sequence.

### *Mar* position in the *hAT* elements phylogeny

**Figure 4:**
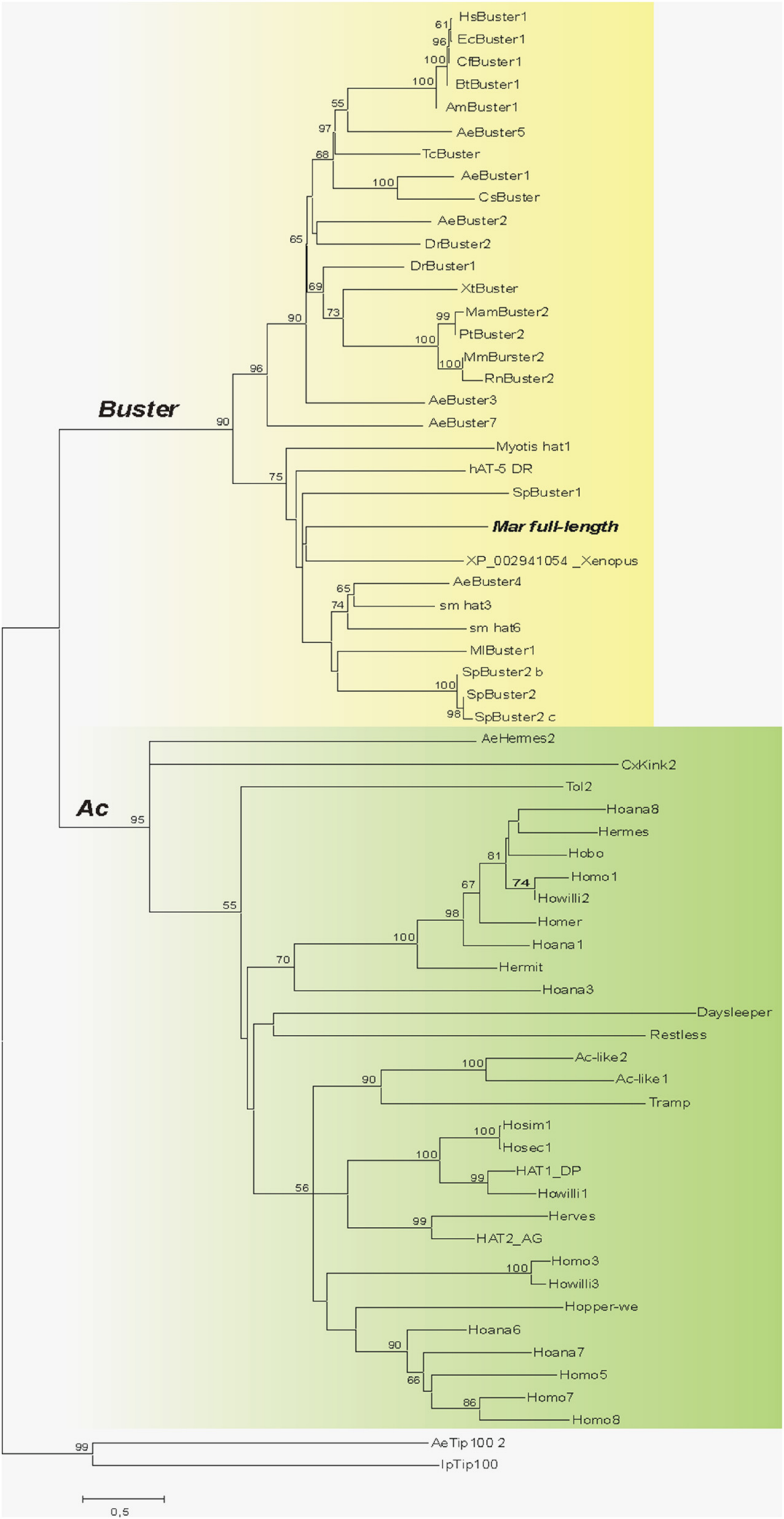
**Neighbor-joining tree showing the relationships among transposase amino acid sequences from several*hAT*elements, including the putative*Mar*transposase*.*** Bootstrap values are shown at the nodes. Values smaller than 50 were omitted. The identity of the sequences can be found in Additional file 9.

To establish the relationship between the *Mar* consensus transposase and the *hAT* superfamily elements, we assembled the transposase sequences described in [17] along with other homologous sequences detected by a BLASTp search. In our analysis, the *hAT *transposase phylogenetic tree also revealed two major clusters of related sequences (Figure 4), previously labeled *Ac* family and *Buster* family [17]. The *Mar* putative transposase fell within the *Buster* family. It forms a clade with *Buster*transposase sequences from bat (*MlBuster1* and *Myotis-hAT1*), mosquito (*AeBuster4*), sea urchin *Strongylocentrotus purpuratus* (*Sp-Buster-1,2,2b,c*), zebrafish *Danio rerio* (*hAT5_DR*) and freshwater planarian *Schmidtea mediterranea* (*sm_hAT3* and *sm_hAT6*). As expected, it is closed to the general transcription factor II-I repeat domain-containing protein 2-like from *X. tropicalis* (XP_002941054). All of the other *Drosophila hAT* elements belong to the *Ac* family. These data confirm that *Mar* belongs to the *Buster* family. 

## Discussion

In *Drosophila* genomes, MITEs are not as abundant and diverse as in mosquitoes and plants. Herein we describe the evolution of *Mar*, a MITE family in *Drosophila*. It is important to note that the designation of MITE is not attributed to a common origin or a taxonomic level in TE classification. The designation of MITE is useful to describe this type of nonautonomous elements that share typical structural features: (1) short elements with no coding capacity, (2) can be present in a high number of copies, (3) contain TIRs, (4) are often located in or near genes and (5) are AT-rich mainly in the inner region [13,19]. The *D. willistoni Mar* element shows these characteristics, but the number of copies is not as high as that of most of the MITE families. However, several MITE families exhibit more modest copy numbers [11,20-24]. We were unable to analyze the number of copies or conservation of TIRs and TSDs in species other than *D. willistoni* from the *willistoni* group; hence we do not know if the *Mar* element spread successfully throughout other genomes. In *D. tropicalis*, no *Mar* MITE copies were found.

*Mar* sequences are present only in *Drosophila* species from the *willistoni* subgroup. In general, the *Mar* phylogeny showed very weak resolution, with a scattered distribution of sequences in different species. This could be indicative of horizontal transfer between species, a common process in TE evolution [22]. However, the species involved are very closely related, and some levels of incongruence were found between different phylogenies of the *willistoni* subgroup, which suggests that saturation, introgression and perhaps incompletely sorted ancestral polymorphisms due to rapid radiation may have occurred [25]. Considering that *Mar* is a multiple copy sequence, the *Mar* phylogeny supports the view that the origin of this MITE occurred after the separation of the *willistoni* and *bocainensis* subgroups, but before the subgroup *willistoni* speciation that began approximately 5.7 Mya [25]. At least in *D. willistoni*, recent transposition bursts have occurred. Some sequences distantly related to *Mar* may be present in species from the *bocainensis* subgroup, as suggested by the Dot blot screening.

In plants and mosquitoes, MITEs are frequently associated with host genes, indicating a potential role for these elements in gene regulation and genome organization [26-28]. We found several *Mar* copies in or near genes in the *D. willistoni* genome. Some of these insertions may be ancient copies present in the ancestor of the *willistoni* subgroup. However, most of the gene-associated copies of *Mar* contain conserved TIRs and TSDs, which suggests that these copies were recently inserted in the *D. willistoni* genome and had no time to accumulate mutations. Because of the recent mobilization of this element, *Mar* is a potentially powerful factor promoting intra- and interspecies variability in the *willistoni* group.

Our analysis revealed that the putative *Mar* transposase is related to the *Buster* family of *hAT* transposons, and the *Mar* element TSD consensus sequence (5^′^-nnnTAnnn-3^′^) also indicates that *Mar* is a *Buster* element. The *Mar* transposase similarity with TFII-I family proteins and transposons from several distinct organisms from divergent taxa raises questions regarding the *Mar* MITE origin. It is known that the *Buster* family consists of both active transposons and domesticated genes that have lost their TIRs but are highly conserved across species [17]. The full-length copy of *Mar* found in *D. willistoni* still retains the TIRs and probably represents an ancient copy of the autonomous *Mar* element rather than a domesticated gene. The intriguing discontinuous distribution of *Buster* family sequences across vertebrates and invertebrates is referred by some authors as a result of horizontal transfer between species [17,29]. More studies are necessary to better understand the relationship between *Mar* and transposons from other species.

Considering the recent mobilization of *Mar* MITEs in *D. willistoni*, we suppose that there should be an active copy allowing the mobilization. Analysis of the coding capacity of the full-length copy of *Mar* suggests that it is no longer active. Thus, it remains uncertain whether this copy was responsible for the recent *Mar* mobility before it became inactive. We cannot exclude the possibility that there is, elsewhere in the *D. willistoni* genome or in other *D. willistoni* strains, a functional copy that could still provide a source of transposase for *Mar* MITE mobilization. Alternatively, another element may provide the transposase for *Mar* mobilization. Cross-mobilization is highly associated with the amplification of MITE families [23]. For instance, in rice, the MITE *mPing* (derived from the autonomous element *Ping*) can be mobilized by the related autonomous element *Pong*[24]. Additionally, another work recently showed cross-mobilization of MITEs from the *Stowaway* family by the *Osmar *transposase [23]. In insects, within the *hAT* superfamily of DNA transposons, cross-mobilization has been reported to the *hobo* element, which is able to mobilize the *hermes* transposon [30]. It would be expected that a *hAT* element would provide the transposase for *Mar* mobilization, since TIR similarity is an essential requirement for MITE transposition [31,32]. de Freitas Ortiz and Loreto [33] characterized five different *hAT* elements in *D. willistoni*, of which three are potentially active. These elements were classified as *Ac* family members [17], and a comparison of their TSD consensus sequences and TIRs with those from the *Mar* element (Additional file 8) does not support the hypothesis that some of these *hAT* elements could be responsible for *Mar* mobilization. The TSD consensus sequences of the *Mar* insertions indicate that they were mobilized by a *Buster* element. To our knowledge, *Mar* is the first *Buster* member described in *Drosophila*; however, more specific searches can identify new *Buster* elements in these species.

The origin of different MITE families is not completely clear, and distinct processes may be involved. One hypothesis is that the MITEs originated by the deletion of autonomous copies [34]. Our results suggest that *Mar* MITEs originated by deletion of a full-length copy and subsequent amplification.

## Conclusions

*Mar* distribution is restricted to the *willistoni* subgroup species and probably originated prior to the diversification of these species. In *D. willistoni*, we found evidence of recent mobilization and amplification. We also identified nonautonomous copies of a full-length *Mar* element in *D. tropicalis* and *D. willistoni*, suggesting that the origin of the *Mar* MITEs may have occurred by internal deletion of an autonomous copy followed by amplification. These elements belong to the *Buster* family and represent the first element of this family identified in *Drosophila*.

## Methods

### *In silico* searches

Searches for *Mar* homologous sequences were conducted in the following genomes using BLASTn on FlyBase: *D. melanogaster*, *D. simulans*, *D. sechellia*, *D. yakuba*, *D. erecta*, *D. ficusphila*, *D. eugracilis*, *D. biarmipes*, *D. takahashii*, *D. elegans*, *D. rhopaloa*, *D. kikkawai*, *D. ananassae*, *D. bipectinata*, *D. pseudoobscura*, *D. persimilis*, *D. willistoni*, *D. mojavensis*, *D. virilis* and *D. grimshawi *[35]. The complete canonical *Mar* sequence (AF518731.1) was used as a query. The presence of conserved TIRs and TSDs in the *Mar* sequences from *D. willistoni* was analyzed by visual inspection of the sequence alignments. We analyzed all hits with an E-value lower than e-100. WebLogo was used for the TSD analysis [36]. Local BLASTn searches were performed against different sequence datasets of the *D. willistoni* genome (coding sequences, intron and gene extended 2,000-bp) to identify *Mar* insertions in gene regions.

### PCR and Dot blot screening

We screened for the presence of *Mar* elements in 61 *Drosophila* species, as well as *Zaprionus indianus*, *Z. tuberculatus*, *Scaptodrosophila latifasciaeformis* and *S. lebanonensis*, using PCR and Dot blotting (Table 1). DNA was extracted from 30 fresh adult flies using a phenol-chloroform protocol [37]. For the PCR reactions, two primers were designed to amplify a *Mar* element fragment of approximately 450 bp: MarF 5^′^-CGCGAATCGTATGTGAA-3^′^ and MarR 5^′^-CGATGTGAGCACGAAGTACA-3^′^ (Figure 1). The PCR reactions (50 μl) were performed as follows: 50 ng of template DNA, 20 pM of each primer, 2.5 mM MgCl_2_ and 1 U *Taq* DNA polymerase. The amplification conditions were as follows: first denaturation at 92°C for 2 minutes, 30 cycles of denaturation at 92°C for 45 seconds, primer annealing at 55°C for 50 seconds and extension at 72°C for 1 minute, followed by extension at 72°C for 5 minutes.

For Dot blot hybridizations, samples of denatured DNA (1 μg) were transferred onto a nylon membrane (Hybond-N+; GE Healthcare Biosciences, Pittsburgh, PA, USA). The AlkPhos Direct Labelling and Detection System and the CDP-Star kit (GE Healthcare) were used to label and detect nucleic acids according to the manufacturer’s instructions. The PCR product of the *Mar* element from *D. willistoni* was used as the probe.

### DNA cloning and sequencing

Amplified samples were visualized on a 0.8% agarose gel. The bands were purified using the GFX Purification Kit (GE Healthcare) and cloned using the TOPO-TA cloning vector (Invitrogen, Carlsbad, CA, USA). Cloned PCR products were sequenced using the universal primers M13 (forward and reverse) on a MegaBACE 500 sequencer. The dideoxy chain-termination reaction was performed using the DYEnamicET kit (GE Healthcare). Two additional primers were used for sequencing the *D. tropicalis* clones: Mar2F 5^′^-CGGACGAAAGGGTATTAACT-3^′^ and Mar2R 5^′^-GCCGTTACACTTGTTTCCTA-3^′^. Both DNA strands were sequenced at least twice or until a reliable sequence was obtained. The sequences from each clone were assembled using Gap4 software from the Stadenpackage [38]. The sequence accession numbers are available in Additional file 2.

### Sequence analysis

Nucleotide and amino acid sequences were aligned using the Muscle tool [39] with default parameters. Nucleotide sequences were used to construct phylogenies according to the following methods: Neighbor-joining and maximum likelihood using the Tamura three-parameter substitution model with a gamma parameter of 2.0 as indicated by model selection analysis. These analyses were implemented using MEGA 5 software [40]. Bayesian analysis was performed using MrBayes 3.1.2 with at least 2,000,000 generations and a burn-in region of 1,000 trees using the Hasegawa, Kishino and Yano (HKY) model with gamma distribution as suggested by the MrModel Test 2.3 program [41]. To calculate the average divergence within and between species, we used MEGA 5 software and the p- distance function [40].

To check whether the full-length *Mar* copies potentially encode a functional transposase, we used FGENESH [42] to predict the existence of coding regions and possible introns. CENSOR software [43] was used to screen query sequences against the reference collection of repeats in Repbase.

The transposase amino acid sequences from several *hAT* superfamily members were compared to the *Mar* consensus sequence from *D. tropicalis*. The protein sequences used were collected based on the work of Arensburger *et al*. [17] from several databases and one manuscript. These sequence identities are shown in Additional file 9. The phylogenetic analysis was conducted using MEGA 5 software [40]. A Neighbor-joining method using the Jones-Taylor-Thornton (JTT) model (with a gamma parameter of 2.0) was used, as indicated by model selection analysis.

## Abbreviations

BLAST: Basic Local Alignment Search Tool; bp: Base pairs; MITE: Miniature inverted-repeat transposable element; PCR: Polymerase chain reaction; TIRs: Terminal inverted repeats; TSD: Target site duplication.

## Competing interests

The authors declare that they have no competing interests.

## Authors’ contributions

MD performed the experiments and assisted in the experimental design. AL designed the study and carried out the *in silico* searches. MD and AL carried out the results analysis and wrote the manuscript. ELSL and VLSV assisted in the experimental design, analysis of results and manuscript writing. VLSV and ELSL provided funding and facilities for the study. All authors read and approved the final manuscript.

## Supplementary Material

Additional file 1 ** Dot blot screening for the presence of *Mar.*** Click here for file

Additional file 2 ** Table describing *Mar* clones obtained in this work from different *Drosophila* strains, along with the nomenclature used, size and accession number in GenBank.** Click here for file

Additional file 3 ** Table describing *Mar* sequences identified in the *D. willistoni* genome with the nomenclature used in this work and the scaffold position.** Click here for file

Additional file 4 ** Genes and putative genes that contain or are near a copy of *Mar.*** Click here for file

Additional file 5 ** Alignment view of the following sequences: canonical *Mar***, ** reconstructed full-length *Mar* (consensus), four *D. tropicalis* clones, four *D. willistoni* copies (scaf72, scaf94, scaf95 and scaf96) and the four primers.** Click here for file

Additional file 6 ** Alignment view of the putative *Mar* transposase and the general transcription factor II-I repeat domain-containing protein 2-like from *Xenopus tropicalis. *** Click here for file

Additional file 7 ** Sequence of the reconstructed full-length *Mar* , its putative coding region and amino acid sequence.** Click here for file

Additional file 8 ** Comparison of the TSD consensus sequences and TIRs from *Mar* and five different *D. willistoni hAT* elements.** Click here for file

Additional file 9 ** Identity and accession number of the sequences used to infer the *hAT* transposase phylogeny. ** Click here for file
