# Supplementary material for: Mar, a MITE family of hAT transposons in Drosophila
Source: Mob DNA. 2012 Aug 31;3:13. doi: 10.1186/1759-8753-3-13 (PMC3517528; doi:10.1186/1759-8753-3-13)
Supplement: Additional file 1 — Dot blot screening for the presence of Mar. [file 1759-8753-3-13-S1.pdf]

Additional file 1

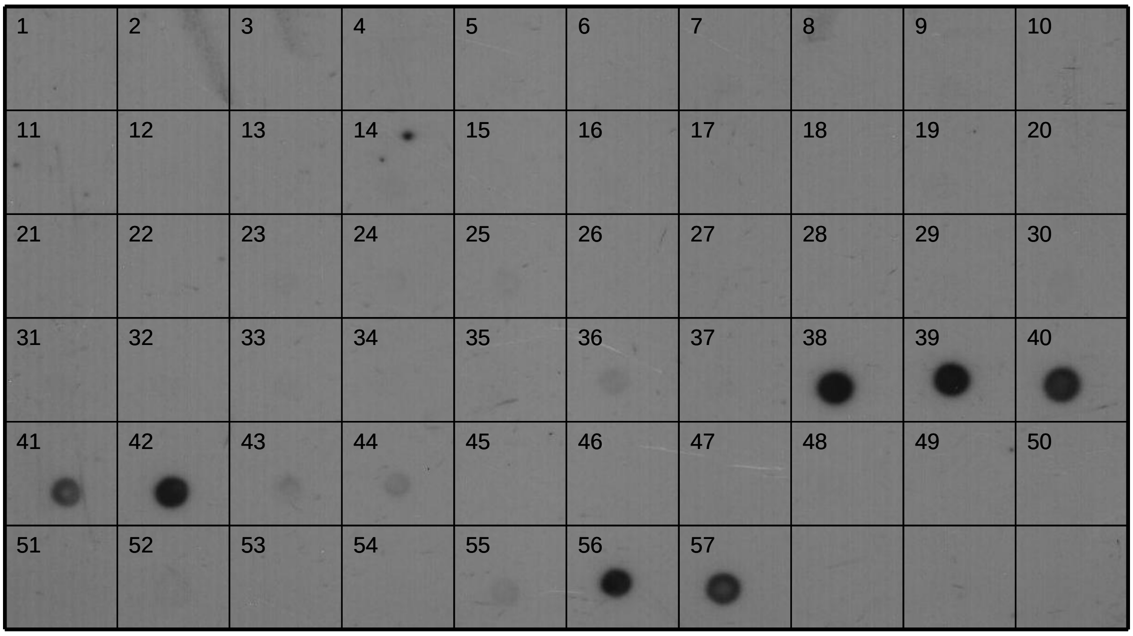

Dot blot screening for the presence of *Mar*. The species tested are the followed: *1 – D. ornatifrons; 2 – D. subbadia; 3 – D. guaru; 4 – D. griseolineata; 5 – D. nappae; 6 – D. paramediostriata; 7 – D. tripunctata; 8 – D. medipictoides; 9 – D. neocardini; 10 – D. polymorpha; 11 – D. ornatipennis; 12 – D. immigrans; 13 – D. funebris; 14 – D. gasici; 15 – D. gaucha; 16 – D. mercatorum; 17 – D. mojavensis; 18 – D. incompta; 19 – D. virilis; 20 – D. robusta; 21 – D. melanogaster; 22 – D. simulans; 23 – D. mauritiana; 24 – D. teissieri; 25 – D. santomea; 26 – D. erecta; 27 – D. yakuba; 28 – D. kikkawai; 29 – D. ananassae; 30 – D. malerkotliana; 31 – D. pseudoobscura; 32 – D. prosaltans; 33 – D. saltans; 34 – D. neoelliptica; 35 – D. sturtevanti; 36 – D. sucinea; 37 – D. nebulosa; 38 - D. willistoni (ww); 39 – D. paulistorum Orinocan; 40 – D. insularis; 41 - D. tropicalis; 42 - D. equinoxialis; 43 - D. capricorni; 44 – D. fumipennis; 45 – D. busckii; 46 – Z. indianus; 47 – Z. tuberculatus; 48 – S. latifasciaeformis; 49 – S. lebanonensis; 50 – without DNA; 51 – D. maculifrons; 52 – D. crocina; 53 – D. hydei; 54 – D. canalinea; 55 – D. orena; 56 – D. willistoni (Wip4); 57 – D. willistoni (17A2)*
