# Supplementary material for: Mar, a MITE family of hAT transposons in Drosophila
Source: Mob DNA. 2012 Aug 31;3:13. doi: 10.1186/1759-8753-3-13 (PMC3517528; doi:10.1186/1759-8753-3-13)
Supplement: Additional file 2 — Table describing Mar clones obtained in this work from different Drosophila strains, along with the nomenclature used, size and accession number in GenBank. [file 1759-8753-3-13-S2.pdf]

Additional file 2

Mar clones obtained in this work from different *Drosophila* strains, along with the nomenclature used, size and accession number at GenBank

**Species/Clone**	**Name**	**Clone size**	**Accession number at GenBank**
Drosophila insularis clone 1	Dinsularis1	365	JQ654744
Drosophila insularis clone 3	Dinsularis3	271	JQ654745
Drosophila insularis clone 5	Dinsularis5	287	JQ654746
Drosophila insularis clone 6	Dinsularis6	287	JQ654747
Drosophila insularis clone 8	Dinsularis8	271	JQ654748
Drosophila paulistorum Andean-Brazilian strain clone 1	Dpaulistorum_and1	359	JQ654749
Drosophila paulistorum Andean-Brazilian strain clone 3	Dpaulistorum_and3	250	JQ654750
Drosophila paulistorum Andean-Brazilian strain clone 4	Dpaulistorum_and4	276	JQ654751
Drosophila paulistorum Andean-Brazilian strain clone 5	Dpaulistorum_and5	276	JQ654752
Drosophila paulistorum Orinocan strain clone 2	Dpaulistorum_ori2	270	JQ654753
Drosophila paulistorum Andean-Brazilian (Rib) strain clone 1	Dpaulistorum_PR1	276	JQ654754
Drosophila paulistorum Andean-Brazilian (Rib) strain clone 2	Dpaulistorum_PR2	268	JQ654755
Drosophila paulistorum Andean-Brazilian (Rib) strain clone 3	Dpaulistorum_PR3	275	JQ654756
Drosophila paulistorum Andean-Brazilian (Rib) strain clone 8	Dpaulistorum_PR8	270	JQ654757
Drosophila paulistorum Andean-Brazilian (Rib) strain clone 9	Dpaulistorum_PR9	270	JQ654758
Drosophila equinoxialis clone 1	Dequinoxialis1	356	JQ654759
Drosophila equinoxialis clone 2	Dequinoxialis2	392	JQ654760
Drosophila equinoxialis clone 3	Dequinoxialis3	506	JQ654761
Drosophila equinoxialis clone 4	Dequinoxialis4	392	JQ654762
Drosophila equinoxialis clone 5	Dequinoxialis5	356	JQ654763
Drosophila willistoni 17A2 strain clone 1	Dwillistoni_17A2_1	351	JQ654764
Drosophila willistoni 17A2 strain clone 5	Dwillistoni_17A2_5	256	JQ654765
Drosophila willistoni White strain clone 5	Dwillistoni_ww5	456	JQ654766
Drosophila willistoni White strain clone 4	Dwillistoni_ww4	459	JQ654767
Drosophila willistoni Wip strain clone 1	Dwillistoni_wip1	418	JQ654768
Drosophila willistoni Wip strain clone 4	Dwillistoni_wip4	456	JQ654769
Drosophila willistoni Wip strain clone 5	Dwillistoni_wip5	418	JQ654770
Drosophila tropicalis clone 7	Dtropicalis7	2482	JQ654771
Drosophila tropicalis clone 8	Dtropicalis8	2487	JQ654772
Drosophila tropicalis clone 10	Dtropicalis10	2482	JQ654773
Drosophila tropicalis clone 16	Dtropicalis16	2489	JQ654774
Drosophila tropicalis clone 17	Dtropicalis17	2428	JQ654775
Drosophila tropicalis clone 29	Dtropicalis29	2483	JQ654776
