# Supplementary material for: Mar, a MITE family of hAT transposons in Drosophila
Source: Mob DNA. 2012 Aug 31;3:13. doi: 10.1186/1759-8753-3-13 (PMC3517528; doi:10.1186/1759-8753-3-13)
Supplement: Additional file 3 — Table describing Mar sequences identified in the D. willistoni genome with the nomenclature used in this work and the scaffold position. [file 1759-8753-3-13-S3.pdf]

Additional file 3

Mar sequences identified in the *D. willistoni* genome with the nomenclature used in this work and the scaffolds position.

Name	Scaffold identification	Begining position	End position
Dwillistoni_scaf1	gnl|dwil|scf2_1100000004958	1032306	1032911
Dwillistoni_scaf2	gnl|dwil|scf2_1100000004963	277589	278102
Dwillistoni_scaf3	gnl|dwil|scf2_1100000004854	77472	76878
Dwillistoni_scaf4	gnl|dwil|scf2_1100000004967	2614439	2615290
Dwillistoni_scaf5	gnl|dwil|scf2_1100000004965	327514	326903
Dwillistoni_scaf6	gnl|dwil|scf2_1100000004595	301019	301594
Dwillistoni_scaf7	gnl|dwil|scf2_1100000004955	122698	123125
Dwillistoni_scaf8	gnl|dwil|scf2_1100000000322	2050	2698
Dwillistoni_scaf9	gnl|dwil|scf2_1100000004949	1493343	1494003
Dwillistoni_scaf10	gnl|dwil|scf2_1100000004620	71409	70759
Dwillistoni_scaf11	gnl|dwil|scf2_1100000004813	65754	65107
Dwillistoni_scaf12	gnl|dwil|scf2_1100000004949	555527	556185
Dwillistoni_scaf13	gnl|dwil|scf2_1100000004521	11928873	11928215
Dwillistoni_scaf14	gnl|dwil|scf2_1100000004521	6540608	6539956
Dwillistoni_scaf15	gnl|dwil|scf2_1100000004359	2912	2291
Dwillistoni_scaf16	gnl|dwil|scf2_1100000005811	640	1290
Dwillistoni_scaf17	gnl|dwil|scf2_1100000004912	25111	24459
Dwillistoni_scaf18	gnl|dwil|scf2_1100000004830	439272	439924
Dwillistoni_scaf19	gnl|dwil|scf2_1100000000768	1401	2035
Dwillistoni_scaf20	gnl|dwil|scf2_1100000004961	1440432	1440857
Dwillistoni_scaf21	gnl|dwil|scf2_1100000004909	608774	609351
Dwillistoni_scaf22	gnl|dwil|scf2_1100000000864	4787	5344
Dwillistoni_scaf23	gnl|dwil|scf2_1100000004909	554976	555584
Dwillistoni_scaf24	gnl|dwil|scf2_1100000004962	576254	575662
Dwillistoni_scaf25	gnl|dwil|scf2_1100000004949	1337602	1336999
Dwillistoni_scaf26	gnl|dwil|scf2_1100000000773	2068	1540
Dwillistoni_scaf27	gnl|dwil|scf2_1100000004962	214088	213482
Dwillistoni_scaf28	gnl|dwil|scf2_1100000005175	1002	217
Dwillistoni_scaf29	gnl|dwil|scf2_1100000004854	340369	340978
Dwillistoni_scaf30	gnl|dwil|scf2_1100000004949	876289	876783
Dwillistoni_scaf31	gnl|dwil|scf2_1100000004590	2633231	2633777
Dwillistoni_scaf32	gnl|dwil|scf2_1100000004949	811236	810887
Dwillistoni_scaf33	gnl|dwil|scf2_1100000004943	3417084	3416495
Dwillistoni_scaf34	gnl|dwil|scf2_1100000003696	5555	4982
Dwillistoni_scaf35	gnl|dwil|scf2_1100000004588	1586	2195
Dwillistoni_scaf36	gnl|dwil|scf2_1100000012097	1151	546
Dwillistoni_scaf37	gnl|dwil|scf2_1100000004514	3235008	3235617
Dwillistoni_scaf38	gnl|dwil|scf2_1100000004954	3533482	3534047
Dwillistoni_scaf39	gnl|dwil|scf2_1100000004954	4150423	4150988
Dwillistoni_scaf40	gnl|dwil|scf2_1100000004822	1962040	1962605
Dwillistoni_scaf41	gnl|dwil|scf2_1100000004887	180585	179976
Dwillistoni_scaf42	gnl|dwil|scf2_1100000004887	429842	429233
Dwillistoni_scaf43	gnl|dwil|scf2_1100000004887	474717	474108
Dwillistoni_scaf44	gnl|dwil|scf2_1100000004887	455657	455048
Dwillistoni_scaf45	gnl|dwil|scf2_1100000004930	44648	44039
Dwillistoni_scaf46	gnl|dwil|scf2_1100000004963	1070515	1069912
Dwillistoni_scaf47	gnl|dwil|scf2_1100000004946	79412	80022
Dwillistoni_scaf48	gnl|dwil|scf2_1100000004949	154792	155219
Dwillistoni_scaf49	gnl|dwil|scf2_1100000004949	1747488	1746879
Dwillistoni_scaf50	gnl|dwil|scf2_1100000004958	117547	116938
Dwillistoni_scaf51	gnl|dwil|scf2_1100000000616	938	1547
Dwillistoni_scaf52	gnl|dwil|scf2_1100000004963	6839	6308
Dwillistoni_scaf54	gnl|dwil|scf2_1100000004963	169074	169645
Dwillistoni_scaf55	gnl|dwil|scf2_1100000004949	1937635	1937176

Dwillistoni_scaf56	gnl|dwil|scf2_1100000004963	4868901	4868330
Dwillistoni_scaf57	gnl|dwil|scf2_1100000004511	1874367	1873796
Dwillistoni_scaf58	gnl|dwil|scf2_1100000004521	11929480	11928909
Dwillistoni_scaf59	gnl|dwil|scf2_1100000004954	5281243	5280672
Dwillistoni_scaf60	gnl|dwil|scf2_1100000004909	981409	980838
Dwillistoni_scaf61	gnl|dwil|scf2_1100000004590	63185	63756
Dwillistoni_scaf62	gnl|dwil|scf2_1100000004558	1682226	1682797
Dwillistoni_scaf63	gnl|dwil|scf2_1100000004884	1040950	1040379
Dwillistoni_scaf64	gnl|dwil|scf2_1100000004852	159424	159995
Dwillistoni_scaf65	gnl|dwil|scf2_1100000004813	68931	68399
Dwillistoni_scaf66	gnl|dwil|scf2_1100000004930	45752	46319
Dwillistoni_scaf67	gnl|dwil|scf2_1100000014301	162	730
Dwillistoni_scaf68	gnl|dwil|scf2_1100000004513	5939298	5938642
Dwillistoni_scaf69	gnl|dwil|scf2_1100000004909	554442	553790
Dwillistoni_scaf70	gnl|dwil|scf2_1100000004909	4924050	4923528
Dwillistoni_scaf71	gnl|dwil|scf2_1100000004423	41528	42184
Dwillistoni_scaf72	gnl|dwil|scf2_1100000004958	725150	722837
Dwillistoni_scaf73	gnl|dwil|scf2_1100000004512	1233768	1234538
Dwillistoni_scaf74	gnl|dwil|scf2_1100000004887	213384	212780
Dwillistoni_scaf75	gnl|dwil|scf2_1100000004958	1471144	1471635
Dwillistoni_scaf76	gnl|dwil|scf2_1100000010275	762	1177
Dwillistoni_scaf77	gnl|dwil|scf2_1100000004138	455	1031
Dwillistoni_scaf78	gnl|dwil|scf2_1100000004654	902	268
Dwillistoni_scaf79	gnl|dwil|scf2_1100000004572	14906	14335
Dwillistoni_scaf80	gnl|dwil|scf2_1100000004970	7527	7936
Dwillistoni_scaf81	gnl|dwil|scf2_1100000004936	55872	55463
Dwillistoni_scaf82	gnl|dwil|scf2_1100000004382	1382947	1383356
Dwillistoni_scaf83	gnl|dwil|scf2_1100000004884	244183	243774
Dwillistoni_scaf84	gnl|dwil|scf2_1100000001947	1341	1750
Dwillistoni_scaf85	gnl|dwil|scf2_1100000004675	27339	27762
Dwillistoni_scaf86	gnl|dwil|scf2_1100000004625	13222	13797
Dwillistoni_scaf87	gnl|dwil|scf2_1100000004949	18742	19396
Dwillistoni_scaf88	gnl|dwil|scf2_1100000011450	43	506
Dwillistoni_scaf89	gnl|dwil|scf2_1100000009372	779	1388
Dwillistoni_scaf90	gnl|dwil|scf2_1100000004888	958313	958736
Dwillistoni_scaf91	gnl|dwil|scf2_1100000004315	1570	1144
Dwillistoni_scaf92	gnl|dwil|scf2_1100000004854	462240	462745
Dwillistoni_scaf93	gnl|dwil|scf2_1100000004954	4923162	4923524
Dwillistoni_scaf94	gnl|dwil|scf2_1100000004884	186050	187777
Dwillistoni_scaf95	gnl|dwil|scf2_1100000004967	4270545	4272453
Dwillistoni_scaf96	gnl|dwil|scf2_1100000011190	6	1676

Note: The first 93 sequences were found in the searches using the canonical *Mar* sequence as query. The sequences 94, 95, 96 and also 72 were found using a *D. tropicalis* clone sequence as query.
