# Supplementary material for: Mar, a MITE family of hAT transposons in Drosophila
Source: Mob DNA. 2012 Aug 31;3:13. doi: 10.1186/1759-8753-3-13 (PMC3517528; doi:10.1186/1759-8753-3-13)
Supplement: Additional file 4 — Genes and putative genes that contain or are near a copy of Mar. [file 1759-8753-3-13-S4.pdf]

Additional file 4

Genes and putative genes that contain or are near a copy of *Mar*.

Gene	Score	e-value	Orthologous	Domains or function
Dwil\GK23646	504	e-142	-	RT
Dwil\GK16085	496	e-139	-	RH, LIM
Dwil\GK10438	496	e-139	-	IG
Dwil\GK21996	480	e-135	+	ecdysone receptor
Dwil\GK16111	470	e-132	+	Utp11, Ufd2P
Dwil\GK12298	456	e-127	+	glutamate receptor
Dwil\GK22081 (mastermind)	446	e-124	+	Transcription coactivator
Dwil\GK18788	446	e-124	+	ubiquitin thiolesterase
Dwil\GK18750 (semaphorin)	446	e-124	+	receptor activity
Dwil\GK17026	446	e-124	-	thioredoxin domain
Dwil\GK23613	203	e-111	-	Flgl
Dwil\GK20297	359	2e-098	-	Piwi-like domain
Dwil\GK18552	353	1e-096	-	Transcription repressor
Dwil\GK10543	307	6e-083	+	microtubule binding
Dwil\GK10422 (chico)	289	1e-077	+	insulin-like growth factor receptor binding
Dwil\GK16644	252	3e-066	-	Importin domain
Dwil\GK23093 (dystroglycan)	186	2e-046	+	Protein binding
Dwil\GK15940	519	e-146	+	gustatory receptor
Dwil\tRNA:GK26087	480	e-134	-	-
Dwil\GK10602	478	e-134	-	Homologue to exon eIF-5A
Dwil\GK21043	468	e-131	-	Gypsy2-I_Dmoj
Dwil\GK23197	446	e-124	-	Unknow
Dwil\GK14562	446	e-124	-	DUF3743
Dwil\GK10515	446	e-124	-	Unknow
Dwil\GK14495	438	e-122	-	Unknow
Dwil\GK10612	385	e-105	-	Unknow
Dwil\GK18774	379	e-104	-	Unknow
Dwil\GK10531 (omega)	353	3e-096	+	dipeptidyl-peptidase activity
Dwil\GK25289	313	3e-084	+	branched-chain-amino-acid transaminase activity
Dwil\GK16590	297	2e-079	-	Unknow
Dwil\tRNA:GK26340	208	1e-052	-	-
Dwil\GK17166	240	4e-062	-	AdoMet_MTase
Dwil\GK25464	230	3e-059	-	Unknow
Dwil\tRNA:GK26349	208	1e-052	-	-
Dwil\GK20176	178	1e-043	-	Unknow
Dwil\GK12334	170	3e-041	+	Unknow

Note: Gene function was checked in the flybase gene database or the conserved domains were examined using Pfam database (Finn *et al*. 2010. Nucleic Acids Research; Database Issue 38:D211-222). In the orthologous column, (+) means presence of orthologous genes in other species and (-) means absence of orthologous genes in other species.
