# Supplementary material for: Mar, a MITE family of hAT transposons in Drosophila
Source: Mob DNA. 2012 Aug 31;3:13. doi: 10.1186/1759-8753-3-13 (PMC3517528; doi:10.1186/1759-8753-3-13)
Supplement: Additional file 5 — Alignment view of the following sequences: canonical Mar, reconstructed full-length Mar (consensus), four D. tropicalis clones, four D. willistoni copies (scaf72, scaf94, scaf95 and scaf96) and the four primers. [file 1759-8753-3-13-S5.pdf]

```
                          *        20         *        40         *        60         *        80         *
Dwilli94  : NNNNNNNCGGCACAAAGAGAGCGCTGAAAAGATGTAGTTGTATGTGTGCGCTTGCTTGTGTGCGTAAGTTGCTTTACACTGCGCAAATTGC :  91
Dwilli95  : CAGGGCCCGGCACAAAGAGAGCGCTGAAAAGATGTAGTAGTATGTGTGCGCTTGCTTGTGTGCGTAAGTAACTTTACACTGCGCAAATTGC :  91
Dwilli96  : -------CGGCACAAAGAGAGCGCTGAAAAGATGTAGTAGTATGTGTGCGCTTGCTTGTGTGCGTAAGTAACTTTACACTGCGCAAATTGC :  84
Mar       : CAGAGGTAGGCACAAAGAGAGCCAGATCAAACTGTCAATGTATGCGTGCGCTTGCTTGTGTACGTAAGCTGCTTTACACTGCGCGAATCGT :  91
Dwilli72  : CAGGGGTAGGCACAAAGAGAGCCAGCTCAAACTGTCAATGTATGCGTGCGATTGCTTGTGTGCGTAAGTATTTG----------------- :  74
Dtropi16  : ------------------------------------------------------------------------------------------- :   -
Dtropi29  : ------------------------------------------------------------------------------------------- :   -
Dtropi10  : ------------------------------------------------------------------------------------------- :   -
Dtropi17  : ------------------------------------------------------------------------------------------- :   -
Dtropi8   : ------------------------------------------------------------------------------------------- :   -
Dtropi7   : ------------------------------------------------------------------------------------------- :   -
consensus : CAGGGGTAGGCACAAAGAGAGCCAGCTCAAACTGTCAATGTATGCGTGCGATTGCTTGTGTGCGTAAGTATTTGTACACTGCGCGAATCGT :  91
MarF      : ---------------------------------------------------------------------------------CGCGAATCGT :  10
MarR      : ------------------------------------------------------------------------------------------- :   -
Mar2F     : ------------------------------------------------------------------------------------------- :   -
Mar2R     : ------------------------------------------------------------------------------------------- :   -

                  100         *       120         *       140         *       160         *       180
Dwilli94  : ATGTGACAAAAGCAAGAAAAATTAGAAA-TGCAATGGAAAACCAATAATTTTGAATTAATATTTTTTTGAAACTCAACTATTTCCTTTA- : 180
Dwilli95  : ATGTGACAAGAGCAAGAAAAATTAGAAA-TGCGATGGAAAACCAATAATTTTGAATTAATATTTTTTTGAAACTCAACTATTTCGTTTAC : 181
Dwilli96  : ATGTGACAAAAGCAAGAAAAATTAGAAA-TGCGATGGAAAACCAATAATTTTGAATTAATATTTTTTTGAAACTCAACTATTTCGTTTAC : 174
Mar       : ATGTGAAGAAACGAATAAAAAAATTAAACTGCAAGTGAAACT------------------------------------------------- : 133
Dwilli72  : -----AAGAAACGAATAAAAAAATTAAACTGCAAGTGAAAAAGAATAATTCCGACCTAATTGTTGTTCAGAAACTCAACTATTTCCTTTAC : 160
Dtropi16  : ----------------------------CTGCAGNGGAAGA----TAATTCCGACCTAATTTTTGTTCAGAAACTCAACTGTTTCCTTTAC :  59
Dtropi29  : ----------------------------CTGCAGTGGAAGA----TAATTCCGACCTAATTTTGGTTCAGAAACTCAACTGTTTCCTTTAC :  59
Dtropi10  : ----------------------------CTGCAGTGGAAGA----TAATTCCGACCTAATTTTTGTTCAGAAACTCAACTGTTTCCTTTAC :  59
Dtropi17  : --------------------------------------------------------------------------------------TTTAC :   5
Dtropi8   : ----------------------------NTGCAGTGGAANA----TAATTCCGACCTAATTTTTGTTCAGAAACTCAACTGTTTCCTTTAC :  59
Dtropi7   : ----------------------------CTGCAGTGGAAGA----TAATTCCGACCTAATTTTTGTTCAGAAACTCAACTGTTTCCTTTAC :  59
consensus : ATGTAAGAAACGAATAAAAAAA-TTAAACTGCAGTGGAAGAAGAATAATTCCGACCTAATTTTTGTTCAGAAACTCAACTGTTTCCTTTAC : 181
MarF      : ATGTGAA------------------------------------------------------------------------------------ :  17
MarR      : ------------------------------------------------------------------------------------------- :   -
Mar2F     : ------------------------------------------------------------------------------------------- :   -
Mar2R     : ------------------------------------------------------------------------------------------- :   -
```

```
                        *       200         *       220         *       240         *       260         *
Dwilli94  : -----TATTATTTGGATAACTTTTTATCCGAAGGAGATCTTCTAAATGGCTATCAGTTAGACTGTTTCGAATAGAACTTTTAATTAATTTC :  266
Dwilli95  : TTCTATATTATTTGGATAACTTTTTATCCGAAGGAGATCTTCTAAATGGCTATCAGTTAGACTGTTTCGAATAGAATTTTTAATTAATTTC :  272
Dwilli96  : TTCTATATTATTTGGATAACTTTTTATCCGAAGGAGATCTTCTAAATGGCTATCAGTTAGACTGTTTCGAATAGAATTTTTAATTAATTTC :  265
Mar       : ------------------------------------------------------------------------------------------- :    -
Dwilli72  : TTCTATATTGTTTGGATACCTTTTTATACGAAGAAGATCTTCTAAATGGCTATCAGTAAGACTATTTCGAATTGAATCTTTAATTAATTTC :  251
Dtropi16  : TTCTATATTGTTTGGATAACTTTTTATACGAAGGAGATCTTCTAAATGGCTATCAGTAAGACTATTTCGAATTGAATCTTTAATTAATTTC :  150
Dtropi29  : TTCTATATTGTTTGGATAACTTTTTATACGAAGGAGATCTTCTAAATGGCTATCAGTAAGACTATTTCGAATTGAATCTTTAATTAATTTC :  150
Dtropi10  : TTCTATATTGTTTGGATAACTTTTTATACGAAGGAGATCTTCTAAATGGCTATCAGTAAGACTATTTCGAATTGAATCTTTAATTAATTTC :  150
Dtropi17  : TTCTATATTGTTTGGATAACTTTTTATACGAAGGAGATCTTCTAAATGGCTATCAGTAAGACTATTTCGAATTGAATCTTTAATTAATTTC :   96
Dtropi8   : TTCTATATTGTTTGGATAACTTTTTATACGAAGGAGATCTTCTAAATGGCTATCAGTAAGACTATTTCGAATTGAATCTTTAATTAATTTC :  150
Dtropi7   : TTCTATATTGTTTGGATAACTTTTTATACGAAGGAGATCTTCTAAATGGCTATCAGTAAGACTATTTCGAATTGAATCTTTAATTAATTTC :  150
consensus : TTCTATATTGTTTGGATAACTTTTTATACGAAGGAGATCTTCTAAATGGCTATCAGTAAGACTATTTCGAATTGAATCTTTAATTAATTTC :  272
MarF      : ------------------------------------------------------------------------------------------- :    -
MarR      : ------------------------------------------------------------------------------------------- :    -
Mar2F     : ------------------------------------------------------------------------------------------- :    -
Mar2R     : ------------------------------------------------------------------------------------------- :    -

                280         *       300         *       320         *       340         *       360
Dwilli94  : ATTGTAGAAAATGTAGTTTCACAAATATATGTTGTGGTGTTTACCACTATTCTATATTTATAGAACATAATTTTAACATTGCCTTTTTGCA :  357
Dwilli95  : ATTGTAGAAAATGTAGTTTCACAAATATAGGTTGTGGT-----------------AAACATAGAACATAATTTTAACATTGCCTTTTTGCA :  346
Dwilli96  : ATTGTAGAAAATGTAGTTTCACAAATATATGTTGTGGT-----------------AAACATAGAACATAATTTTAACATTGCCTTTTTGCA :  339
Mar       : ------------------------------------------------------------------------------------------- :    -
Dwilli72  : ATTGTCGAAAATGTGGATTCGCAAATGTATGTTGTGGT-----------------AAACATGGAAGATAATTTTAACATTGCCTTTTTGGA :  325
Dtropi16  : ATTGTCGAAAATGTGGATTCGCAAATGTATGTTGTGGT-----------------AAACATGGAAGATAATTTTAACATTGCCTTTTTAGA :  224
Dtropi29  : ATTGTCGAAAATGTGGATTCGCAAATGTATGTTGTGGT-----------------AAACATGGAAGATAATTTTAACATTGCCTTTTTAGA :  224
Dtropi10  : ATTGTCGAAAATGTGGATTCGCAAATGTATGTTGTGGT-----------------AAACATGGAAGATAATTTTAACATTGCCTTTTTAGA :  224
Dtropi17  : ATTGTCGAAAATGTGGATTCGCAAATGTATGTTGTGGT-----------------AAACATGGAAGATAATTTTAACATTGCCTTTTTAGA :  170
Dtropi8   : ATTGTCGAAAATGTGGATTCGCAAATGTATGTTGTGGT-----------------AAACATGGAAGATAATTTTAACATTGCCTTTTTAGA :  224
Dtropi7   : ATTGTCGAAAATGTGGATTCGCAAATGTATGTTGTGGT-----------------AAACATGGAAGATAATTTTAACATTGCCTTTTTAGA :  224
consensus : ATTGTCGAAAATGTGGATTCGCAAATGTATGTTGTGGT-----------------AAACATGGAAGATAATTTTAACATTGCCTTTTTAGA :  346
MarF      : ------------------------------------------------------------------------------------------- :    -
MarR      : ------------------------------------------------------------------------------------------- :    -
Mar2F     : ------------------------------------------------------------------------------------------- :    -
Mar2R     : ------------------------------------------------------------------------------------------- :    -
```

```
                  *        380         *        400         *        420         *        440         *
Dwilli94  : AAACGGAAATTTATCTATCGGTATTTCTTGCCAAAATTCTATTCCACTACTTA-----GCGGCAGCATCAAGTCAGTTTTTATGACCTGTA :  443
Dwilli95  : AACCGGAAATTTATCTATCGCGATTTCTCGCCAAAATTCTATTCCACTACTTA-----GCGGCAGCATCAAGTCAGTTTTCATGACCTGTA :  432
Dwilli96  : AACCGGAAATTTATCTATCGCGATTTCTCGCCAAAATTCTATTCCACTACTTA-----GCGGCAGCATCAAGTCAGTTTTCATGACCTGTA :  425
Mar       : ------------------------------------------------------------------------------------------- :    -
Dwilli72  : ATTAGGAAATTTATCCATGGGAATTTCTCGCCAAAATTCTATTCCACTACTTATGTTAGTGGCAGCATCAAGTCAGTTTTCATGACCTGTA :  416
Dtropi16  : ATTAGGAAATTTATCCATAGGAATGTTTCGCCAAAATTCTATTCCACTACTTA-----GTGGCAGCATCAAGTCAGTTTTCATGACCTGTA :  310
Dtropi29  : ATTAGGAAATTTATCCATAGGAATGTTTCGCCAAAATTCTATTCCACTACTTA-----GTGGCAGCATCAAGTCAGTTTTCATGACCTGTA :  310
Dtropi10  : ATTAGGAAATTTATCCATAGGAATGTTTCGCCAAAATTCTATTCCACTACTTA-----GTGGCAGCATCAAGTCAGTTTTCATGACCTGTA :  310
Dtropi17  : ATTAGGAAATTTATCCATAGGAATGTTTCGCCAAAATTCTATTCCACTACTTA-----GTGGCAGCATCAAGTCAGTTTTCATGACCTGTA :  256
Dtropi8   : ATTAGGAAATTTATCCATAGGAATGTTTCGCCAAAATTCTATTCCACTACTTA-----GTGGCAGCATCAAGTCAGTTTTCATGACCTGTA :  310
Dtropi7   : ATTAGGAAATTTATCCATAGGAATGTTTCGCCAAAATTCTATTCCACTACTTA-----GTGGCAGCATCAAGTCAGTTTTCATGACCTGTA :  310
consensus : ATTAGGAAATTTATCCATAGGAATGTTTCGCCAAAATTCTATTCCACTACTTA-----GTGGCAGCATCAAGTCAGTTTTCATGACCTGTA :  432
MarF      : ------------------------------------------------------------------------------------------- :    -
MarR      : ------------------------------------------------------------------------------------------- :    -
Mar2F     : ------------------------------------------------------------------------------------------- :    -
Mar2R     : ------------------------------------------------------------------------------------------- :    -

               460         *        480         *        500         *        520         *        540
Dwilli94  : TTTCCTCCCTAATTTGTTTTGGAATTGACTCAGATTCTGCTTCTAGGGGGTTATTAAAAACTGCAATGAGCCAATTGATTTGTTGAAAATC :  534
Dwilli95  : TTTCCTCCCTAATTTCTTTTGGAATTGACTCAGATTCTGCTTCTAGGGGGTTATTAAAAACTGCAATGAGCCAATTGATTTGTTAAAAATC :  523
Dwilli96  : TTTCCTCCCTAATTTCTTTTGGAATTGACTCAGATTCTGCTTCTAGGGGGTTATTAAAAACTGCAATGAGCCAATTGATTTGTTGAAAATC :  516
Mar       : ------------------------------------------------------------------------------------------- :    -
Dwilli72  : TTTCGTTCTTAATTTCTTCTGGGATTAATTCTGATTCTGCTTCAAGGGGGCTATTGAAAACTGCGATTAGCCAATTGATTTGTTTAAAATC :  507
Dtropi16  : TTTCCTTCTTAATTTCTTCTGGGATTAATTCTGATTCTGCTTCAAGGGGGCTATTGAAAACTGCGATTAGCCAATTGATTTGTTTAAAATC :  401
Dtropi29  : TTTCCTTCTTAATTTCTTCTGGGATTAATTCTGATTCTGCTTCAAGGGGGCTATTGAAAACTGCGATTAGCCAATTGATTTGTTTAAAATC :  401
Dtropi10  : TTTCCTTCTTAATTTCTTCTGGGATTAATTCTGATTCTGCTTCAAGGGGGCTATTGAAAACTGCGATTAGCCAATTGATTTGTTTAAAATC :  401
Dtropi17  : TTTCCTTCTTAATTTCTTCTGGGATTAATTCTGATTCTGCTTCAAGGGGGCTATTGAAAACTGCGATTAGCCAATTGATTTGTTTAAGATC :  347
Dtropi8   : TTTCCTTCTTAATTTCTTCTGGGATTAATTCTGATTCTGCTTCAAGGGGGCTATTGAAAACTGCGATTAGCCAATTGATTTGTTTAAAATC :  401
Dtropi7   : TTTCCTTCTTAATTTCTTCTGGGATTAATTCTGATTCTGCTTCAAGGGGGCTATTGAAAACTGCGATTAGCCAATTGATTTGTTTAAAATC :  401
consensus : TTTCCTTCTTAATTTCTTCTGGGATTAATTCTGATTCTGCTTCAAGGGGGCTATTGAAAACTGCGATTAGCCAATTGATTTGTTTAAAATC :  523
MarF      : ------------------------------------------------------------------------------------------- :    -
MarR      : ------------------------------------------------------------------------------------------- :    -
Mar2F     : ------------------------------------------------------------------------------------------- :    -
Mar2R     : ------------------------------------------------------------------------------------------- :    -
```

```
                 *         560         *         580         *         600         *         620         *
Dwilli94  : AGAAAATTTTTCCGCTATTTCAGAGGAAAGCGTATTAATTGTCTCTGCGTATTCCTGAATTCGGGAAGGGTTGGGGTGCAATGTGGCAATT : 625
Dwilli95  : AGAAAATTTTTCCGCTATTTCAGAGGAAAGGGTATTAACTGCCTCTGCGTATTCCTGAATTCGGGAAGGGTTGGGGTGCAATGTAGCAATT : 614
Dwilli96  : AGAAAATTTTTCCGCTATTTCAGAGGAAAGGGTATTAACTGCCTCTGCGTATTCCTGAATTCGGGAAGGGTTGGGGTGCAATGTAGCAATT : 607
Mar       : ------------------------------------------------------------------------------------------- : -
Dwilli72  : AGAAAATTTTTCAGCTATTTCGGACGAAAGGGTATTAACTGCCTCAGCGTATTCCTGAATTCGGGAAGGGTTAGGGTACAATGTTACACTT : 598
Dtropi16  : AGAAAATTTTTCAGCTATTTCGGACGAAAGGGTATTAACTGCCTCAGCGTATTCCTGAATTCGGGAAGGGTTAGGGTACAATGTTACACTT : 492
Dtropi29  : AGAAAATTTTTCAGCTATTTCGGACGAAAGGGTATTAACTGCCTCAGCGTATTCCTGAATTCGGGAAGGGTTAGGGTACAATGTTACACTT : 492
Dtropi10  : AGAAAATTTTTCAGCTATTTCGGACGAAAGGGTATTAACTGCCTCAGCGTATTCCTGAATTCGGGAAGGGTTAGGGTACAATGTTACACTT : 492
Dtropi17  : AGAAAATTTTTCAGCTATTTCGGACGAAAGGGTATTAACTGCCTCAGCGTATTCCTGAATTCGGGAAGGGTTAGGGTACAATGTTACACTT : 438
Dtropi8   : AGAAAATTTTTCAGCTATTTCGGACGAAAGGGTATTAACTGCCTCAGCGTATTCCTGAATTCGGGAAGGGTTAGGGTACAATGTTACACTT : 492
Dtropi7   : AGAAAATTTTTCAGCTATTTCGGACGAAAGGGTATTAACTGCCTCAGCGTATTCCTGAATTCGGGAAGGGTTAGGGTACAATGTTACACTT : 492
consensus : AGAAAATTTTTCAGCTATTTCGGACGAAAGGGTATTAACTGCCTCAGCGTATTCCTGAATTCGGGAAGGGTTAGGGTACAATGTTACACTT : 614
MarF      : ------------------------------------------------------------------------------------------- : -
MarR      : ------------------------------------------------------------------------------------------- : -
Mar2F     : ---------------------CGGACGAAAGGGTATTAACT-------------------------------------------------- : 20
Mar2R     : ------------------------------------------------------------------------------------------- : -

            640         *         660         *         680         *         700         *         720
Dwilli94  : TCTGCAGTTCGCTGAAGGCTGGACAGGTCATTGTTTTTTATTTGAATTTTAAGACCATTTATTTTGCAAGTAAATTTATTGATTGCTGCAA : 716
Dwilli95  : TCTGCAGTTCGCTGAAGGCTGGACAGGTAATTGTTTTTTATTTGAATTTTAAGACCATTTATTTTGCAGGTAAATTTATTGATTGCTGCAA : 705
Dwilli96  : TCTGCAGTTCGCTGAAGGCTGGACAGGTCATTGTTTTTTATTTGAATTTTAAGACCATTTATTTTGCAGGTAAATTTATTGATTGCTGCAA : 698
Mar       : ------------------------------------------------------------------------------------------- : -
Dwilli72  : TCTGCAGTTCCCTGAAGACTGGACAGGTCATTTTTTTTGATCTGAATTTGAAGACTATTAATTTTGCGAGTAAAATTTTGGATCGCCGCAA : 689
Dtropi16  : TCTGCAGTTCGCTGAAGGCTGGACAGGTCATTATTTTTCATCTGAATTTGAAGACTACTAATTTTGCGAGTAAAATTTTTTATCGCTGCAA : 583
Dtropi29  : TCTGCAGTTCGCTGAAGGCTGGACAGGTCATTATTTTTCATCTGAATTTGAAGACTACTAATTTTGCGAGTAAAATTTTTTATCGCTGCAA : 583
Dtropi10  : TCTGCAGTTCGCTGAAGGCTGGACAGGTCATTATTTTTCATCTGAATTTGAAGACTACTAATTTTGCGAGTAAAATTTTTTATCGCTGCAA : 583
Dtropi17  : TCTGCAGTTCGCTGAAGGCTGGACAGGTCATTATTTTTCATCTGAATTTGAAGACTACTAATTTTGCGAGTAAAATTTTTTATCGCTGCAA : 529
Dtropi8   : TCTGCAGTTCGCTGAAGGCTGGACAGGTCATTATTTTTCATCTGAATTTGAAGACTACTAATTTTGCGAGTAAAATTTTTTATCGCTGCAA : 583
Dtropi7   : TCTGCAGTTCGCTGAAGGCTGGACAGGTCATTATTTTTCATCTGAATTTGAAGACTACTAATTTTGCGAGTAAATTTTTTTATCGCTGCAA : 583
consensus : TCTGCAGTTCGCTGAAGGCTGGACAGGTCATTATTTTTCATCTGAATTTGAAGACTACTAATTTTGCGAGTAAAATTTTTTATCGCTGCAA : 705
MarF      : ------------------------------------------------------------------------------------------- : -
MarR      : ------------------------------------------------------------------------------------------- : -
Mar2F     : ------------------------------------------------------------------------------------------- : -
Mar2R     : ------------------------------------------------------------------------------------------- : -
```

```
               *      740       *      760       *      780       *      800       *      82
Dwilli94  : TTAAATCGGACGGAAGTTTGTTGCGGCCTTGAAGCTGAAGATTTAATTGATTAATGAAAAGGACTATCTCAGTTAGAAA----------CG :  797
Dwilli95  : TTAAATCGGACGGAAGTTTGTTGCGGCCTTGAAGCTGAAGATTTAATTGATTAATGAAAAGGACTATGACAGTTAGAAA----------CG :  786
Dwilli96  : TTAAATCGGACGGAAGTTTGTTGCGGCCTTGAAGCTGAAGATTTAATTGATTAATGAAAAGGACTATGTCAGTTAGAAAAAATAATGGATT :  789
Mar       : ------------------------------------------------------------------------------------------- :    -
Dwilli72  : TTAATTCGGACGGA----------------------------------------------------------------------------- :  703
Dtropi16  : TTAAATCGGACGGAAGTTTATTGCGGCCCTGAAGCTGAAGATTTAATTGATTAATAAAAAGAACTAAATCAGTTAGAAA----------TG :  664
Dtropi29  : TTAAATCGGACGGAAGTTTATTGCGGCCCTGAAGCTGAAGATTTAATTGATTAGTAAAAAGAACTAAATCAGTTAGAAA----------TG :  664
Dtropi10  : TTAAATCGGACGGAAGTTTATTGCGGCCCTGAAGCTGAAGATTTAATTGATTAATAAAAAGAACTAAATCAGTTAGAAA----------TG :  664
Dtropi17  : TTAAATCGGACGGAAGTTTATTGCGGCCCTGAAGCTGAAGATTTAATTGATTAATAAAAAGAACTAAATCAGTTAGAAA----------TG :  610
Dtropi8   : TTAAATCGGACGGAAGTTTATTGCGGCCCTGAAGCTGAAGATTTAATTGATTAATAAAAAGAACTAAATCAGTTAGAAA----------TG :  664
Dtropi7   : TTAAATCGGACGGAAGTTTATTGCGGCCCTGAAGCTGAAGATTTAATTGATTAATAAAAAGAACTAAATCAGTTAGAAA----------TG :  664
consensus : TTAAATCGGACGGAAGTTTATTGCGGCCCTGAAGCTGAAGATTTAATTGATTAATAAAAAGAACTAAATCAGTTAGAAA----------TG :  786
MarF      : ------------------------------------------------------------------------------------------- :    -
MarR      : ------------------------------------------------------------------------------------------- :    -
Mar2F     : ------------------------------------------------------------------------------------------- :    -
Mar2R     : ------------------------------------------------------------------------------------------- :    -

            0         *      840       *      860       *      880       *      900       *
Dwilli94  : CCAAGTCTAAAATAAATAATGGATTTTTTAAAGAAGCTAACAATTCTATATCTTTTTCTAAGTTCTCGCTTTCTAAGAA-----TTACTTC :  883
Dwilli95  : CCAAGTCTAAAATAAATAATGGATTTTTTAAAGAAGCTAACAATTCTTTATCTTTTTCTAAGTTCTCGCTTTCTAAGAATGAAATTACTTC :  877
Dwilli96  : TTAAGTCTAAAATAAATAATGGATTTTTTAAAGAAGCTAACAATTCTTTATCTTTTTCTAAGTTCTCGCTTTCTAAGAATGAAATTACTTC :  880
Mar       : ------------------------------------------------------------------------------------------- :    -
Dwilli72  : ------------------------------------------------------------------------------------------- :    -
Dtropi16  : CCAAGTCTAAAATAAATAATGGATTTTTTAAAGAAGTTAATAATTCTTTATCCTTTTCTAAATTTTCCTTTTCTAAGAAAGAAATTACTTC :  755
Dtropi29  : CCAAGTCTAAAATAAATAATGGATTTTTTAAAGAAGTTAATAATTCTTTATCCTTTTCTAAATTTTCCTTTTCTAAGAAAGAAATTACTTC :  755
Dtropi10  : CCAAGTCTAAAATAAATAATGGATTTTTTAAAGAAGTTAATAATTCTTTATCCTTTTCTAAATTTTCCTTTTCTAAGAAAGAAATTACTTC :  755
Dtropi17  : CCAAGTCTAAAATAAATAATGGATTTTTTAAAGAAGTTAATAATTCTTTATCCTTTTCTAAATTTTCCTTTTCTAAGAAAGAAATTACTTC :  701
Dtropi8   : CCAAGTCTAAAATAAATAATGGATTTTTTAAAGAAGTTAATAATTCTTTATCCTTTTCTAAATTTTCCTTTTCTAAGAAAGAAATTACTTC :  755
Dtropi7   : CCAAGTCTAAAATAAATAATGGATTTTTTAAAGAAGTTAATAATTCTTTATCCTTTTCTAAATTTTCCTTTTCTAAGAAAGAAATTACTTC :  755
consensus : CCAAGTCTAAAATAAATAATGGATTTTTTAAAGAAGTTAATAATTCTTTATCCTTTTCTAAATTTTCCTTTTCTAAGAAAGAAATTACTTC :  877
MarF      : ------------------------------------------------------------------------------------------- :    -
MarR      : ------------------------------------------------------------------------------------------- :    -
Mar2F     : ------------------------------------------------------------------------------------------- :    -
Mar2R     : ------------------------------------------------------------------------------------------- :    -
```

```
                   920         *       940         *       960         *       980         *      1000
Dwilli94  : ATTACGAAGAGTATATAGTCTGTCTAAACTTTGGCCTCGGCTCAGCCACCTAACATCAGTAAAAAGGCAAAGATCCTCATATTCAGCGCCG :  974
Dwilli95  : ATTTCAAAGAGTGTATAATCTGTCTAAACTTTGGCCTCGGCTCAGCCACCTAACATCAGTAAAAAGGCAAAGATCCTCATATTCAGCGCCG :  968
Dwilli96  : ATTTCGAAGAGTGTATAATCTGTCTAAACTTTGGCCTCGGCTCAGCCACCTAACATCAGTAAAAAGGCAAAGATCCTCATATTCAGCGCCG :  971
Mar       : ------------------------------------------------------------------------------------------- :    -
Dwilli72  : ------------------------------------------------------------------------------------------- :    -
Dtropi16  : TTTACGAAGACTGTATAGTCTGTCCAAACTTTGGCCACGGCTCAGCCACCTAACATCAGTAAAAAGGCTAAGATCCTTATATTCAGCGCCG :  846
Dtropi29  : TTTACGAAGGCTGTATAGTCTGTCCAAACTTTGGCCACGGCTCAGCCACCTAACATCAGTAAAAAGGCTAAGATCCTTATATTCAGCGCCG :  846
Dtropi10  : TTTACGAAGACTGTATAGTCTGTCCAAACTTTGGCCACGGCTCAGCCACCTAACATCAGTAAAAAGGCTAAGATCCTTATATTCAGCGCCG :  846
Dtropi17  : TTTACGAAGACTGTATAGTCTGTCCAAACTTTGGCCACGGCTCAGCCACCTAACATCAGTAAAAAGGCTAAGATCCTTATATTCAGCGCCG :  792
Dtropi8   : TTTACGAAGACTGTATAGTCTGTCCAAACTTTGGCCACGGCTCAGCCACCTAACATCAGTAAAAAGGCTAAGATCCTTATATTCAGCGCCG :  846
Dtropi7   : TTTACGAAGACTGTATAGTCTGTCCAAACTTTGGCCACGGCTCAGCCACCTAACATCAGTAAAAAGGCTAAGATCCTTATATTCAGCGCCG :  846
consensus : TTTACGAAGACTGTATAGTCTGTCCAAACTTTGGCCACGGCTCAGCCACCTAACATCAGTAAAAAGGCTAAGATCCTTATATTCAGCGCCG :  968
MarF      : ------------------------------------------------------------------------------------------- :    -
MarR      : ------------------------------------------------------------------------------------------- :    -
Mar2F     : ------------------------------------------------------------------------------------------- :    -
Mar2R     : ------------------------------------------------------------------------------------------- :    -

                    *      1020         *      1040         *      1060         *      1080         *
Dwilli94  : AATTCTGAGAGAAAAGATTTAAACTTTCTATGGGTTAGCGAATTATGACCACCTTTAATGCGATTAATAATTCTAATTGCTACACCGATTG : 1065
Dwilli95  : AATTCTGAGAGAAAAGATTTAAACTTTCTATGGGTTAGCGAATTATGGCCACCTTTAATGCGATTAATAATTCTAATTGCTACACCCATTG : 1059
Dwilli96  : AATTCTGAGAGAAAAGATTTAAACTTTCTATGGGTTAGCGAATTATGGCCACCTTTAATGCGATTAATAATTCTAATTGCTACACCCATTG : 1062
Mar       : ------------------------------------------------------------------------------------------- :    -
Dwilli72  : ------------------------------------------------------------------------------------------- :    -
Dtropi16  : ATTTCTACGAGAAAGGATTTAAACTTTCTATGGGTTAGCGAATTATGACCACCTTTTATGCGATTAATAATTTTAGTTGCTACACCCATTG :  937
Dtropi29  : ATTTCTACGAGAAAGGATTTAAACTTTCTATGGGTTAGCGAATTATGACCACCTTTTATGCGATTAATAATTTTAGTTGCTACACCCATTG :  937
Dtropi10  : ATTTCTACGAGAAAGGATTTAAACTTTCTATGGGTTAGCGAATTATGACCACCTTTTATGCGATTAATAATTTTAGTTGCTACACCCATTG :  937
Dtropi17  : ATTTCTACGAGAAAGGATTTAAACTTTCTATGAGTTAGCGAATTATGACCACCTTTTATGCGATTAATAATTTTAGTTGCTACACCCATTG :  883
Dtropi8   : ATTTCTACGAGAAAGGATTTAAACTTTCTATGGGTTAGCGAATTATGACCACCTTTTATGCGATTAATAATTTTAGTTGCTACACCCATTG :  937
Dtropi7   : ATTTCTACGAGAAAGGATTTAAACTTTCTATGGGTTAGCGAATTATGACCACCTTTTATGCGATTAATAATTTTAGTTGCTACACCCATTG :  937
consensus : ATTTCTACGAGAAAGGATTTAAACTTTCTATGGGTTAGCGAATTATGACCACCTTTTATGCGATTAATAATTTTAGTTGCTACACCCATTG : 1059
MarF      : ------------------------------------------------------------------------------------------- :    -
MarR      : ------------------------------------------------------------------------------------------- :    -
Mar2F     : ------------------------------------------------------------------------------------------- :    -
Mar2R     : ------------------------------------------------------------------------------------------- :    -
```

```
                       1100         *        1120         *        1140         *        1160         *        1180
Dwilli94  : CATCCACAACTTGAAA-----------AACAATGCTTGC--TGATGGATGAAACAATGGAAAAATGGTATTTATCCTACATGTCTTTTGAC : 1143
Dwilli95  : CATCCACAACTTGAAAAATTTTT-GCAAACAATGCTTGC--TGATGGATGAAACAATGGAAAAATGGTATTTCTCCTACATGTCTATTGAC : 1147
Dwilli96  : CATCCACAACTTGAAAAATTTTT-GCAAACAATGCTTGC--TGATGGATGAAACAATGGAAAAATGGTATTTCTCCTACATGTCTATTGAC : 1150
Mar       : ------------------------------------------------------------------------------------------- :    -
Dwilli72  : -----------------------------------------TGATGAATAGA--------------------------------------- :  714
Dtropi16  : CATCAACAACTTGAAACATTTTTTGTGAACAATGCGTGCGCTGGTGGAAGAAACAGTGGAAAAATGGTATTTCTCCTATTTTTCTATTGGC : 1028
Dtropi29  : CATCAACAACTTGAAACATTTTT-GCAAACAATGCTTGC--TGATGTATGAAACAATGGAAAAATGGTATTTCTCCTATTTTTCTATTGGC : 1025
Dtropi10  : CATCAACAACTTGAAACATTTTT-GCAAACAATGCTTGC--TGATGGATGAAACAATGGAAAAATGGTATTTCTCCTATTTTTCTATTGGC : 1025
Dtropi17  : CATCAACAACTTGAAACATTTTT-GCAAACAATGCTTGC--TGATGGATGAAACAATGGAAAAATGGTATTTCTCCTATTTTTCTATTGGC :  971
Dtropi8   : CATCAACAACTTGAAACATTTTT-GCAAACAATGCTTGC--TGATGGATGAAACAATGGAAAAATGGTATTTCTCCTATTTTTCTATTGGC : 1025
Dtropi7   : CATCAACAACTTGAAACATTTTT-GCAAACAATGCTTGC--TGATGGATGAAACAATGGAAAAATGGTATTTCTCCTATTTTTCTATTGGC : 1025
consensus : CATCAACAACTTGAAACATTTTT-GCAAACAATGCTTGC--TGATGGATGAAACAATGGAAAAATGGTATTTCTCCTATTTTTCTATTGGC : 1147
MarF      : ------------------------------------------------------------------------------------------- :    -
MarR      : ------------------------------------------------------------------------------------------- :    -
Mar2F     : ------------------------------------------------------------------------------------------- :    -
Mar2R     : ------------------------------------------------------------------------------------------- :    -

                    *        1200         *        1220         *        1240         *        1260         *
Dwilli94  : ATG-GCTAAGAAATCCTTCATTCTTTCCCTTCATGGTGGCGGCACCATCAGTGCAAATTGAGGACATTTTATTT----GATAATTGGAATG : 1229
Dwilli95  : ATG-GCCAAGAAATCCTTCATTCTTTCCCTTCATGGTGGCGGCACCGTCAGTGCAAATTGAGGACATTTTATTT----GCTAATTGGAATG : 1233
Dwilli96  : ATG-GCCAAGAAATCCTTCATTCTTTCCCTTCATGGTGGCGGCACCGTCAGTGCAAATTGAGGACATTTTATTT----GCTAATTGGAATG : 1236
Mar       : ------------------------------------------------------------------------------------------- :    -
Dwilli72  : ----------------------------------------------------------TGATGACATTTTATCT----GTCATTTGGAGTG :  743
Dtropi16  : ATGGACAAAGAAATCCTTCATTGTTTCCTTTCATGGTGGCGGCACCGTCAGTACAAATTGATGAGATTTTATCTATCTGTCAATTGGAGTG : 1119
Dtropi29  : ATG-ACCAAGAAATCCTTCATTGTTTCCTTTCATGGTGGCGGCACCGTCAGTACAAATTGATGAGATTTTATCT----GTCAATTGGAGTG : 1111
Dtropi10  : ATG-ACCAAGAAATCCTTCATTGTTTCCTTTCATGGTGGCGGCACCGTCAGTACAAATTGATGAGATTTTATCT----GTCAATTGGAGTG : 1111
Dtropi17  : ATG-ACCAAGAAATCCTTCATTGTTTCCTTTCATGGTGGCGGCACCGTCAGTACAAATTGATGAGATTTTATCT----GTCAATTGGAGTG : 1057
Dtropi8   : ATG-ACCAAGAAATCCTTCATTGTTTCCTTTCATGGTGGCGGCACCGTCAGTACAAATTGATGAGATTTTATCT----GTCAATTGGAGTG : 1111
Dtropi7   : ATG-ACCAAGAAATCCTTCATTGTTTCCTTTCATGGTGGCGGCACCGTCAGTACAAATTGATGAGATTTTATCT----GTCAATTGGAGTG : 1111
consensus : ATG-ACCAAGAAATCCTTCATTGTTTCCTTTCATGGTGGCGGCACCGTCAGTACAAATTGATGAGATTTTATCT----GTCAATTGGAGTG : 1233
MarF      : ------------------------------------------------------------------------------------------- :    -
MarR      : ------------------------------------------------------------------------------------------- :    -
Mar2F     : ------------------------------------------------------------------------------------------- :    -
Mar2R     : ------------------------------------------------------------------------------------------- :    -
```

```
               1280         *      1300         *      1320         *      1340         *      1360
Dwilli94  : AAAGTACATGCTT-CTCAAAGGCGTCAAACGTACTTTTTCCATTTACATGACTGTGAAAAGAATGTAATTTTAAAAATTCATCGCAAATGT : 1319
Dwilli95  : AAAGTACATGCTT-CTCAAAGGCATCAAACGTACTTTTTCCATTTACATGACTGTGAAAAGAATGTAACTTTAAAAATTCATCGCAAATGT : 1323
Dwilli96  : AAAGTACATGCTT-CTCAAAGGCATCAAACGTACTTTTTCCATTTACATGACTGTGAAAAGAATGTAACTTTAAAAATTCATCGCAAATGT : 1326
Mar       : ------------------------------------------------------------------------------------------ :    -
Dwilli72  : AAAGTACATGCTT-CTCAAAATCATCAAAAGTGCTTGTCCCATTCACATGACCATGAAAGGAATGCAATTTTAGAAATTCATCACAGATGT :  833
Dtropi16  : AAAGTACATGCTT-CTCAAAAGCATCAAAAGTGCTTTTCCCATTCACATGACCATGAAAGGAATGCAGTTTTAGAAACTCGTCACAGATGT : 1209
Dtropi29  : AAAGTACATGCTT-CTCAAAAGCATCAAAAGTGCTTTTCCCATTCACATGACCATGAAAGGAATGCAGTTTTAGAAACTCATCACAGATGT : 1201
Dtropi10  : AAAGTACATGCTT-CTCAAA-GCATCAAAAGTGCTTTTCCCATTCACATGACCATGAAAGGAATGCAGTTTTAGAAACTCATCACAGATGT : 1200
Dtropi17  : AAAGTACATGCTTTCTCAAAAGCATCAAAAGTGCTTTTCCCATTCACATGACCATGAAAGGAATGCAGTTTTAGAAACTCATCACAGATGT : 1148
Dtropi8   : AAAGTACATGCTT-CTCAAAAGCATCAAAAGTGCTTTTCCCATTCACATGACCATGAAAGGAATGCAGTTT-AGAAACTCATCACAGATGT : 1200
Dtropi7   : AAAGTACATGCTT-CTCAAAAGCATCAAAAGTGCTTTTCCCATTCACATGACCATGAAAGGAATGCAGTTT-AGAAACTCATCACAGATGT : 1200
consensus : AAAGTACATGCTT-CTCAAAAGCATCAAAAGTGCTTTTCCCATTCACATGACCATGAAAGGAATGCAGTTTTAGAAACTCATCACAGATGT : 1323
MarF      : ------------------------------------------------------------------------------------------ :    -
MarR      : ------------------------------------------------------------------------------------------ :    -
Mar2F     : ------------------------------------------------------------------------------------------ :    -
Mar2R     : ------------------------------------------------------------------------------------------ :    -

                 *      1380         *      1400         *      1420         *      1440         *
Dwilli94  : TAAAGTCGTAATCAATAG-TCCTAACACAAATAACCATTTGACTTATATTTCTAACATCTACGCTTTCATCCAAGCAAATTGAAAAGAAGG : 1409
Dwilli95  : TAAAGTCGTAATCAATAG-TCCTAACACAAATAACCATTTGACTAATATTTCTAACATCTACGCTTTCATCCAAACAAATTGAAAAGAAGG : 1413
Dwilli96  : TAAAGTCGTAATCAATAG-TCCTAACACAAATAACCATTTGACTAATATTTCTAACATCTACGCTTTCATCCAAACAAATTGAAAAGAAGG : 1416
Mar       : ------------------------------------------------------------------------------------------ :    -
Dwilli72  : TGAATTCGTAATCTATGG-TTCTGACACAAATAATCATTTGACTAATATTTCTAATATCTACACTTCCATCTAAACAAACTGAAAAGAAAG :  923
Dtropi16  : TGAATTCGTAATCTATGGGTTCTGACACAAATAATCATTTGACTAATATTTCTAATATCTACACTTTCATCTAAACAAACTGAAAAGAAAG : 1300
Dtropi29  : TGAATTCGTAATCTATGG-TTCTGACACAAATAATCATTTGACTAATATTTCTAATATCTACACTTTCATCTAAACAAACTGAAAAGAAAG : 1291
Dtropi10  : TGAATTCGTAATCTATGG-TTCTGACACAAATAATCATTTGACTAATATTTCTAATATCTACACTTTCATCTAAACAAACTGAAAAGAAAG : 1290
Dtropi17  : TGAATTCGTAATCTATGG-TTCTGACACAAATAATCATTTGACTAATATTTCTAATATCTACACTTTCATCTAAACAAACTGAAAAGAAAG : 1238
Dtropi8   : TGAATTCGTAATCTATGG-TTCTGACACAAATAATCATTTGACTAATATTTCTAATATCTACACTTTCATCTAAACAAACTGAAAAGAAAG : 1290
Dtropi7   : TGAATTCGTAATCTATGGGTTCTGACACAA-TAATCATTTGACTAATATTTCTAATATCTACACTTTCATCTAAACAAACTGAAAAGAAAG : 1290
consensus : TGAATTCGTAATCTATGG-TTCTGACACAAATAATCATTTGACTAATATTTCTAATATCTACACTTTCATCTAAACAAACTGAAAAGAAAG : 1413
MarF      : ------------------------------------------------------------------------------------------ :    -
MarR      : ------------------------------------------------------------------------------------------ :    -
Mar2F     : ------------------------------------------------------------------------------------------ :    -
Mar2R     : ------------------------------------------------------------------------------------------ :    -
```

```
             1460        *      1480        *      1500        *      1520        *      1540
Dwilli94  : CACATCCCTCTATTCTTTTTTTAGGTTCTGAGTGTAAG-TATTGGCCTATTTCCTTGCTCCTGCGAGTTATAGTTCGACTTGATAAAGGAG : 1499
Dwilli95  : CACAGCCCTCTATTCTTTTTTTAGTTTCTGAGTATAAG-TATTGGCCTATTTCTTCGGTCCTGCGAGTTATAGTTCGACTTGATAAAGGAG : 1503
Dwilli96  : CACAGCCCTCTATTCTTTTTTTAGTTTCTGAGTATAAG-TATTGGCCTATTTCTTCGGTCCTGCGAGTTATAGTTCGACTTGATAAAGGAG : 1506
Mar       : ------------------------------------------------------------------------------------------- :    -
Dwilli72  : CACAGCCCTCTATTCGTTGTTTAGTTTGTGAGTATAAG-TATTGCCCTATTTCTTTGGTCCTGCGAGTTATTGTTCTATTCGACAGTGGAA : 1013
Dtropi16  : CACAGCC-TCTATTCGTTGGTTAG-TTGTGAGTATAAA-TATTGCCCTACTTCTTCAGTC-TGCGAGTTATTGTTCTATTCGACAGTGGAA : 1387
Dtropi29  : CACAGCCCTCTATTCATTGGTTAGTTTGTGAGTATAAA-TATTGCCCTACTTCTTCAGTCCTGCGAGTTATTGTTCTATTCGACAGTGGAA : 1381
Dtropi10  : CACAGCCCTCTATTCGTTGGTTAGTTTGTGAGTATAAA-TATTGCCCTACTTCTTCAGTCCTGCGAGTTATTGTTCTATTCGACAGTGGAA : 1380
Dtropi17  : CACAGCC-TCTATTCGTTGGTTAGTTTGTGAGTATAAA-TATTGCCCTACTTCTTCAGTC-TGCGAGTTATTGTACTATTCGACAGTGGAA : 1326
Dtropi8   : CACAGCCCTCTATTCGTTGGTTAGTTTGTGAGTATAAA-TATTGCCCTACTTCTTCAGTCCTGCGAGTTATTGTTCTATTCGACAGTGGAA : 1380
Dtropi7   : CACAGCC-TCTATTCGTTGGTTAGTTTGTGAGTATAAAATATTGCCCTACTTCTTCAGTC-TGCGAGTTATTGTTCTATTCGACAGTGGAA : 1379
consensus : CACAGCCCTCTATTCGTTGGTTAGTTTGTGAGTATAAA-TATTGCCCTACTTCTTCAGTCCTGCGAGTTATTGTTCTATTCGACAGTGGAA : 1503
MarF      : ------------------------------------------------------------------------------------------- :    -
MarR      : ------------------------------------------------------------------------------------------- :    -
Mar2F     : ------------------------------------------------------------------------------------------- :    -
Mar2R     : ------------------------------------------------------------------------------------------- :    -

               *      1560        *      1580        *      1600        *      1620        *     16
Dwilli94  : TATCGTTTATTAAACTATAAGACTCCTGGTCCCTATGCCCCAAAATATCAAGCACCTCCAAGC--AAAGGTCTTTAAAAAACATACCGTCT : 1588
Dwilli95  : TATCGTTTATTAAACTATAGGACTCCTGGTCCCTATGCCCCAAAATATCAAGCACCTCCAAGC--AAAGGTCTTTAAAAAACATACCGTCT : 1592
Dwilli96  : TATCGTTTATTAAACTATAGGACTCCTGGTCCCTATGCCCCAAAATATCAAGCACCTCCAAGC--AAAGGTCTTTAAAAAACATACCGTCT : 1595
Mar       : ------------------------------------------------------------------------------------------- :    -
Dwilli72  : TATCCTTAATCAAACTGTATGACTCTTTCTCCTTTTGGCTCAAAATATCGAGGATCTCTAAAC--AAAGGTCTGCAAAAAACATACCATCT : 1102
Dtropi16  : TATCCTTAATCAAACTGTATGACTCTTTCTCCTTTTGGCCCAAAATATCGAGGATCTCTAAAC--AAAGGTCTTTAAAAAACATGCCATCT : 1476
Dtropi29  : TATCCTTAATCAAACTGTATGACTCTTTCTCCTTTTGGCCCAAAATATCGAGGATCTCTAAAC--AAAGGTCTTTAAAAAACATGCCATCT : 1470
Dtropi10  : TATCCTTAATCAAACTGTATGACTCTTTCTCCTTTTGGCCCAAAATATCGAGGATCTCTAAAC--AAAGGTCTTTAAAAAACATGCCATCT : 1469
Dtropi17  : TATCCTTAATCAAACTGTATGACTCTTTCTCCTTTTGGCCCAAAATATCGAGGATCTCTAAAC--AAAGGTCTTTAAAAAACATGCCATCT : 1415
Dtropi8   : TATCCTTAATCAAACTGTATGACTCTTTCTCCTTTTGGCCCAAAATATCGAGGATCTCTAAAC-AAAAGGTCTTTAAAAAACATGCCATCT : 1470
Dtropi7   : TATCCTTAATCAAACTGTATGACTCTTTCTC-TTTTGGCCCAAAATATCGAGGATCTCTAAAACAAAAGGTCTTTAAAAAACATGCCATCT : 1469
consensus : TATCCTTAATCAAACTGTATGACTCTTTCTCCTTTTGGCCCAAAATATCGAGGATCTCTAAAC--AAAGGTCTTTAAAAAACATGCCATCT : 1592
MarF      : ------------------------------------------------------------------------------------------- :    -
MarR      : ------------------------------------------------------------------------------------------- :    -
Mar2F     : ------------------------------------------------------------------------------------------- :    -
Mar2R     : ------------------------------------------------------------------------------------------- :    -
```

```
                40         *       1660         *       1680         *       1700         *       1720
Dwilli94  : TCGAA--TGGGCGGCATTTTC-GAGCTAGTGCTAGTGCAATGGTAAAAGACTCTTT-TAATTGCACATTTTCGGAGTCACTGTCTGGACCG : 1675
Dwilli95  : TCGAA--TGGGCGGCATTTTC-GAGCTAATGCTAGTGCAATGGCAAAAGACGCTTT-TAATTGCACATTTTCCGAGTTACTATCTGGACCG : 1679
Dwilli96  : TCGAA--TGGGCGGCATTTTC-GAGCTAATGCTAGTGCAATGGCAAAAGACGCTTT-TAATTGCACATTTTC------------------- : 1663
Mar       : ------------------------------------------------------------------------------------------- :    -
Dwilli72  : TTAAA--TGGGCGACCTTTCC-GTGCTAATGCTATTGCAACAGCAAAAGAAGCTTT-CAATATAACATTTTCATCGACACTATGTCCGGCA : 1189
Dtropi16  : TTAAA--TGGGCGACCTTTCC-GTGCTAATGCTATTGCAACAGCAAAAGAAGCTTT-CAATATAACATTTTCATCGACGCTATGTCCGGCA : 1563
Dtropi29  : TTAAA--TGGGCGACCTTTCC-GTGCTAATGCTATTGCAACAGCAAAAGAAGCTTT-CAATATAACATTTTCATCGACGCTATGTCCGGCA : 1557
Dtropi10  : TTAAA--TGGGCGACCTTTCC-GTGCTAATGCTATTGCAACAGCAAAAGAAGCTTT-CAATATAACATTTTCATCGACGCTATGTCCGGCA : 1556
Dtropi17  : TTAAA--TGGGCGACCTTTCC-GTGCTAATGCTATTGCAACAGCAAAAGAAGCTTT-CAATATAACATTTTCATCGACGCTATGTCCGGCA : 1502
Dtropi8   : TTAAAATGGGGCGACCTTTCCCGTGCTAATGCTATTGCAACAGCAAAAGAAGCTTTTCAATATAACATTTTCATCGACGCTATGTCCGGCA : 1561
Dtropi7   : TTAAA--TGGGGGACCTTTCC-GTGCTAATGCTATTGCAACAGCAAAAGAAGCTTT-CAATATAACATTTTCATCGACGCTATGTCCGGCA : 1556
consensus : TTAAA--TGGGCGACCTTTCC-GTGCTAATGCTATTGCAACAGCAAAAGAAGCTTT-CAATATAACATTTTCATCGACGCTATGTCCGGCA : 1679
MarF      : ------------------------------------------------------------------------------------------- :    -
MarR      : ------------------------------------------------------------------------------------------- :    -
Mar2F     : ------------------------------------------------------------------------------------------- :    -
Mar2R     : ------------------------------------------------------------------------------------------- :    -

            *       1740         *       1760         *       1780         *       1800         *       1820
Dwilli94  : TTTTCTATAACAGATTGGCGAACTCTATTGTTTTTGAGATAAAACAAACGTTTAAG----------------------------------- : 1731
Dwilli95  : TTTTCTATAACAGGTTGGCGATCTCTATTGTTTTTGAGATAAAACAAACGTTTAAGTTTATGAAATAAATTTAATCTTTCGTTTTCGTTTT : 1770
Dwilli96  : ------------------------------------------------------------------------------------------- :    -
Mar       : ------------------------------------------------------------------------------------------- :    -
Dwilli72  : TGGCTTGGGGTGCAATTGCGGAGCCTACTGATTTTACTGTTAAATAAACTATTAAGTTTTTGAAAAATTTCTAGTTTTTCATCTTCATTTT : 1280
Dtropi16  : TGGCTTGGGGTGAAATTGCGGAACCTACTGATTTTACTGTTAAATAAACTATTAAGTTTTTGAAAAATTTCTAGTTTTTCATCTTCATTTT : 1654
Dtropi29  : TGGCTTGGGGTGAAATTGCGGAACCTACTGATTTTACTGTTAAATAAACTATTAAGTTTTTGAAAAATTTCTAGTCTTTCATCTTCATTTT : 1648
Dtropi10  : TGGCTTGGGGTGAAATTGCGGAACCTACTGATTTTACTGTTAAATAAACTATTAAGTTTTTGAAAAATTTCTAGTTTTTCATCTTCATTTT : 1647
Dtropi17  : TGGCTTGGGGTGAAATTGCGGAACCTACTGATTTTACTGTTAAATAAACTATTAAGTTTTTGAAAAATTTCTAGTTTTTCATCTTCATTTT : 1593
Dtropi8   : TGGCTTGGGGTGAAATTGCGGAACCTACTGATTTTACTGTTAAATAAACTATTAAGTTTTTGAAAAATTTCTAGTTTTTCATCTTCATTTT : 1652
Dtropi7   : TGGCTTGGGGTGAAATTGCGGAACCTACTGATTTTACTGTTAAATAAACTATTAAATTTTTGAAAAATTTCTAGTTTTTCATCTTCATTTT : 1647
consensus : TGGCTTGGGGTGAAATTGCGGAACCTACTGATTTTACTGTTAAATAAACTATTAAGTTTTTGAAAAATTTCTAGTTTTTCATCTTCATTTT : 1770
MarF      : ------------------------------------------------------------------------------------------- :    -
MarR      : ------------------------------------------------------------------------------------------- :    -
Mar2F     : ------------------------------------------------------------------------------------------- :    -
Mar2R     : ------------------------------------------------------------------------------------------- :    -
```

```
                   *      1840         *      1860         *      1880         *      1900         *
Dwilli94  : ------------------------------------------------------------------------------------------- :    -
Dwilli95  : TAATCTGGGACTCGTTATGAGTG---GAAAAGTGACGATTAAGGTGGAATTTATATTTTTTGGCAATTACTCGATGACATATTAAACATTC : 1858
Dwilli96  : ------------------------------------------------------------------------------------------- :    -
Mar       : ------------------------------------------------------------------------------------------- :    -
Dwilli72  : TGTTGTGCAACTTTTTGTGAATGCTAACAAAGTGCCGTTGAATGCAATATTTAGATATTTTGGTAATCACCCGATGACAAACTA------- : 1364
Dtropi16  : TGTTGTGCAACTCTTTGTGAATGCTAACAAAGTGCCGTTGAATGCAATATTTAGATATTTTGGTAATCACCCGATTACAAACTATGCATTC : 1745
Dtropi29  : TGTTGTGCAACTCTTTGTGAATGCTAACAAAGTGCCGTTGAATGCAATATTTAGATATTTTGGTAATCACCCGATTACAAACTATGCATTC : 1739
Dtropi10  : TGTTGTGCAACTCTTTGTGAATGCTAACAAAGTGCCGTTGAATGCAATATTTAGATATTTTGGTAATCACCCGATTACAAACTATGCATTC : 1738
Dtropi17  : TGTTGTGCAACTCTTTGTGAATGCTAACAAAGTGCCGTTGAATGCAATATTTAGATATTTTGGTAATCACCCGATTACAAACTATGCATTC : 1684
Dtropi8   : TGTTGTGCAACTCTTTGTGAATGCTAACAAAGTGCCGTTGAATGCAATATTTAGATATTTTGGTAATCACCCGATTACAAACTATGCATTC : 1743
Dtropi7   : TGTTGTGCAACTCTTTGTGAATGCTAACAAAGTGCCGTTGAATGCAATATTTAGATATTTTGGTAATCACCCGATTACAAACTATGCATTC : 1738
consensus : TGTTGTGCAACTCTTTGTGAATGCTAACAAAGTGCCGTTGAATGCAATATTTAGATATTTTGGTAATCACCCGATTACAAACTATGCATTC : 1861
MarF      : ------------------------------------------------------------------------------------------- :    -
MarR      : ------------------------------------------------------------------------------------------- :    -
Mar2F     : ------------------------------------------------------------------------------------------- :    -
Mar2R     : ------------------------------------------------------------------------------------------- :    -

               1920         *      1940         *      1960         *      1980         *      2000
Dwilli94  : ------------------------------------------------------------------------------------------- :    -
Dwilli95  : TGCCTCATTGTTCAAATTTAATC--CAAAAAAAAATTGGTTTTCCCATTCGAC-------------------------------------- : 1909
Dwilli96  : ------------------------------------------------------------------------------------------- :    -
Mar       : ------------------------------------------------------------------------------------------- :    -
Dwilli72  : -------TTGCTAATATTTAATAATTAAAAAAGAACTGCTTTTCCCAATTAACATTCCACATGACTTAGCTATAGTTTTAATAAACGTGTC : 1448
Dtropi16  : TGGCTTATTGCTAATATTTAACA--TAAAAAAGAACTGCTCTTCCCAATTAACATTCCACATGACTTAGCTATAGTTTTAATAAACGTGTC : 1834
Dtropi29  : TGGCTTATTGCTAATATTTAACA--TAAAAAAGAACTGCTTTTCCCAATTAACATTCCACATGACTTAGCTATAGTTTTAATAAACGTGTC : 1828
Dtropi10  : TGGCTTATTGCTAATATTTAACA--TAAAAAAGAACTGCTTTTCCCAATTAACATTCCACATGACTTAGCTATAGTTTTAATAAACGTGTC : 1827
Dtropi17  : TGGCTTATTGCTAATATTTAACA--TAAAAAAGAACTGCTTTTCCCAATTAACATTCCACATGACTTAGCTATAGTTTTAATAAACGTGTC : 1773
Dtropi8   : TGGCTTATTGCTAATATTTAACA--TAAAAAAGAACTGCTTTTCCCAATTAACATTCCACATGACTTAGCTATAGTTTTAATAAACGTGTC : 1832
Dtropi7   : TGGCTTATTGCTAATATTTAACA--TAAAAAAGAACTGCTTTTCCCAATTAACATTCCACATGACTTAGCTATAGTTTTAATAAACGTGTC : 1827
consensus : TGGCTTATTGCTAATATTTAACA--TAAAAAAGAACTGCTTTTCCCAATTAACATTCCACATGACTTAGCTATAGTTTTAATAAACGTGTC : 1950
MarF      : ------------------------------------------------------------------------------------------- :    -
MarR      : ------------------------------------------------------------------------------------------- :    -
Mar2F     : ------------------------------------------------------------------------------------------- :    -
Mar2R     : ------------------------------------------------------------------------------------------- :    -
```

```
                 *      2020         *      2040         *      2060         *      2080         *
Dwilli94  : ------------------------------------------------------------------------------------------ :    -
Dwilli95  : ------------------------------------------------------------------------------------------ :    -
Dwilli96  : ------------------------------------------------------------------------------------------ :    -
Mar       : ------------------------------------------------------------------------------------------ :    -
Dwilli72  : AAAGCAACAACAA--AAATACCAAAAAAAATTTGGAAACAAATTAGAAGCAAAGAACAAAGCCAAAGTCATTAAAAATACTATTTAAATTT : 1537
Dtropi16  : AAAGCAACAACAAGAAAATACCACAAAATTTTTGAAAACAAATTAGAAGCAAAGAACAAAGCCAAAGTCTTTAAAAAGACCATTTGAATTT : 1925
Dtropi29  : AAAGCAACAACAAGAAAATACCACAAAATTTTTGAAAACAAATTAGAAGCAAAGAACAAAGCCAAAGTCTTTAGAAAGACCATTTGAATTT : 1919
Dtropi10  : AAAGCAACAACAAGAAAATACCACAAAATTTTTGAAAATAAATTAGAAGCAAAGAACAGAGCCGAAGTCTTTAAAAAGACCATTTGAATTT : 1918
Dtropi17  : AAAGCAACAACAAGAAAATACCACAAAATTTTTGAAAACAAATTAGAAGCAAAGAACAAAGCCAAAGTCTTTAAAAAGACCATTTGAATTT : 1864
Dtropi8   : AAAGCAACAACAAGAAAATACCACAAAATTTTTGAAAACAAATTAGAAGCAAAGAACAAAGCCAAAGTCTTTAAAAAGACCATTTGAATTT : 1923
Dtropi7   : AAAGCAACAACAAGAAAATACCACAAAATTTTTGAAAACAAATTAGAAGCAAAGAACAAAGCCAAAGTCTTTAAAAAGACCATTTGAATTT : 1918
consensus : AAAGCAACAACAAGAAAATACCACAAAATTTTTGAAAACAAATTAGAAGCAAAGAACAAAGCCAAAGTCTTTAAAAAGACCATTTGAATTT : 2041
MarF      : ------------------------------------------------------------------------------------------ :    -
MarR      : ------------------------------------------------------------------------------------------ :    -
Mar2F     : ------------------------------------------------------------------------------------------ :    -
Mar2R     : ------------------------------------------------------------------------------------------ :    -

              2100         *      2120         *      2140         *      2160         *      2180
Dwilli94  : --------------------------CACT------------------------------------------------------------ : 1735
Dwilli95  : ------------------------------------------------------------------------------------------ :    -
Dwilli96  : ------------------------------------------------------------------------------------------ :    -
Mar       : ------------------------------------------------------------------------------------------ :    -
Dwilli72  : TAACAAGAATAAACGCAATACCACAACACTTGTTACAACTTGGAAGGTCAAGTGAAACTAAATCGCGAGAAAATCTCGCGTTCCCAAACAT : 1628
Dtropi16  : TAACAAGAATAAACACAATGCCACACCACTTGTTACAACTTGGAAGGTCAAGTGAAACTAAATCCCGCGAAAATATCGCGTTCCCAAACAT : 2016
Dtropi29  : TAACAAGAATAAACACAATGCCACACCACTTGTTACAACTTGGAAGGTCAAGTGAAACTAAATCCCGCGAAAATATCGCGTTCCCAAACAT : 2010
Dtropi10  : TAACAAGAATAAACACAATGCCACACCACTTGTTACAACTTGGAAGGTCAAGTGAAACTAAATCCCGCGAAAATATCGCGTTCCCAAACAT : 2009
Dtropi17  : TAACAAGAATAAACACAATGCCACACCACTTGTTACAACTTGGAAGGTCAAGTGAAACTAAATCCCGCGAAAATATCGCGTTCCCAAACAT : 1955
Dtropi8   : TAACAAGAATAAACACAATGCCACACCACTTGTTACAACTTGGAAGGTCAAGTGAAACTAAATCCCGCGAAAATATCGCGTTCCCAAACAT : 2014
Dtropi7   : TAACAAGAATAAACACAATGCCACACCACTTGTTACAACTTGGAAGGTCAAGTGAAACTAAATCCCGCGAAAATATCGCGTTCCCAAACAT : 2009
consensus : TAACAAGAATAAACACAATGCCACACCACTTGTTACAACTTGGAAGGTCAAGTGAAACTAAATCCCGCGAAAATATCGCGTTCCCAAACAT : 2132
MarF      : ------------------------------------------------------------------------------------------ :    -
MarR      : ------------------------------------------------------------------------------------------ :    -
Mar2F     : ------------------------------------------------------------------------------------------ :    -
Mar2R     : ------------------------------------------------------------------------------------------ :    -
```

```
                 *      2200         *      2220         *      2240         *      2260         *
Dwilli94   : ------------------------------------------------------------------------------------------- :    -
Dwilli95   : ------------------------------------------------------------------------------------------- :    -
Dwilli96   : --------------CGAGTTAC--------------------------------------------------------------------- : 1671
Mar        : ------------------------------------------------------------------------------------------- :    -
Dwilli72   : CATTGACCATTTGCAAAAAATGCATGGTAGGTAGCAAATTGTACAAAAAAAAATTTAATGTTCGCAGTTTTGCGCAATAGATTCTGCAATC : 1719
Dtropi16   : CATTGACCATTTGCAAAAAATGCATGGTAGGTAGCAAAT---------------------------------------AGATTCTGCAATC : 2068
Dtropi29   : CATTGACCATTTGCAAAAAATGCATGGTAGGTAGCAAAT---------------------------------------AGATTCTGCAATC : 2062
Dtropi10   : CATTGACCATTTGCAAAAAATGCGTGGTAGGTAGCAAAT---------------------------------------AGATTCTGCAATC : 2061
Dtropi17   : CATTGACCATTTGCAAAAAATGCATGGTAGGTAGCAAAT---------------------------------------AGATTCTGCAATC : 2007
Dtropi8    : CATTGACCATTTGCAAAAAATGCATGGTAGGTAGCAAAT---------------------------------------AGATTCTGCAATC : 2066
Dtropi7    : CATTGACCATTTGCAAAAAATGCATGGTAGGTAGCAAAT---------------------------------------AGATTCTGCAATC : 2061
consensus  : CATTGACCATTTGCAAAAAATGCATGGTAGGTAGCAAATTGTACAAAAAAAAATTTAATGTTCGCAGTTTTGCGCAATAGATTCTGCAATC : 2223
MarF       : ------------------------------------------------------------------------------------------- :    -
MarR       : ------------------------------------------------------------------------------------------- :    -
Mar2F      : ------------------------------------------------------------------------------------------- :    -
Mar2R      : ------------------------------------------------------------------------------------------- :    -

              2280         *      2300         *      2320         *      2340         *      2360
Dwilli94   : ------------------------------------------------------------------------------------------- :    -
Dwilli95   : ------------------------------------------------------------------------------------------- :    -
Dwilli96   : ------------------------------------------------------------------------------------------- :    -
Mar        : ------------------------------------------------------------------------------------------- :    -
Dwilli72   : CTTTCTACCTACTATACAAGTTATTTAAATATCAAAAAAAAAAAACAAGTGCCGGGTT-TCTTTTCTATAGGAAACAAGTGTAACGGCCGC : 1809
Dtropi16   : TTTTCTACCTACTATACAAGTTATTTAAATAT---AAAAAAAAAACAAATGCCGGTCTATTTTTTCTATAGGAAACAAGTGTAACGGCCGC : 2156
Dtropi29   : TTTTCTACCTACTATACAAGTTATTTAAATAT---AAAAAAAAAACAAATGCCGGTCTATTTTTTCTATAGGAAACAAGTGTAACGGCCGC : 2150
Dtropi10   : TTTTCTACCTACTATACAAGTTATTTAAATAT---AAAAAAAAAACAAATGCCGGTCTATTTTTTCTATAGGAAACAAGTGTAACGGCCGC : 2149
Dtropi17   : TTTTCTACCTACTATACAAGTTATTTAAATAT---AAAAAAAAAACAAATGCCGGTCTATTTTTTCTATAGGAAACAAGTGTAACGGCCGC : 2095
Dtropi8    : TTTTCTACCTACTATACAAGTTATTTAAATAT---AAAAAAGAAACAAATGCCGGTCTATTTTTTCTATAGGAAACAAGTGTAACGGCCGC : 2154
Dtropi7    : TTTTCTACCTACTATACAAGTTATTTAAATAT---AAAAAAAAAACAAATGCCGGTCTATTTTTTCTATAGGAAACAAGTGTAACGGCCGC : 2149
consensus  : TTTTCTACCTACTATACAAGTTATTTAAATATCAAAAAAAAAAAACAAATGCCGGTCTATTTTTTCTATAGGAAACAAGTGTAACGGCCGC : 2314
MarF       : ------------------------------------------------------------------------------------------- :    -
MarR       : ------------------------------------------------------------------------------------------- :    -
Mar2F      : ------------------------------------------------------------------------------------------- :    -
Mar2R      : --------------------------------------------------------------------TAGGAAACAAGTGTAACGGC--- :   20
```

```
               *      2380         *      2400         *      2420         *      2440         *      2
Dwilli94  : ------------------------------------------------------------------------------------------- :    -
Dwilli95  : ------------------------------------------------------------------------------------------- :    -
Dwilli96  : ------------------------------------------------------------------------------------------- :    -
Mar       : --------------------------------GCGGAATTTTCCCACAACCCTTTTTTACCGAGTCGCCTTAAAAAACAAAGTACAGTCAT :  192
Dwilli72  : TACCTGTAATAGGAAATATTTGAGTATGCCAAGCGGAATTTTCCCACAACCCTTTTTTACCGAATCGCCTTAAAAAACAAAGTACAGTCAT : 1900
Dtropi16  : TACCTGCAATAGGAAATATTTGAGTATGTCAAGCGGAATTTTCCCACAACCCTTTTTTACCGAATCGCCTTAAAAAACAAAGTA--GTCAT : 2245
Dtropi29  : TACCTGCAATAGGAAATATTTGAGTATGTCAAGCGGAATTTTCCCACAACCCTTTTTTACCGAATCGCCTTAAAAAACAAAGTA--GTCAT : 2239
Dtropi10  : TACCTGCAATAGGAAATATTTGAGTATGTCAAGCGGAATTTTCCCACAACCCTTTTTTACCGAATCGCCTTAAAAAACAAAGTA--GTCAT : 2238
Dtropi17  : TACCTGCAATAGGAAATATTTGAGTATGTCAAGCGGAATTTTCCCACAACCCTTTTTTACCGAATCGCCTTAAAAAACAAAGTA--GTCAT : 2184
Dtropi8   : TACCTGCAATAGGAAATATTTGAGTATGTCAAGCGGAATTTTCCCACAACCCTTTTTTACCGAATCGCCTTAAAAAACAAAGTA--GTCAT : 2243
Dtropi7   : TACCTGCAATAGGAAATATTTGAGTATGTCAAGCGGAATTTTCCCACAACCCTTTTTTACCGAATCGCCTTAAAAAACAAAGTA--GTCAT : 2238
consensus : TACCTGCAATAGGAAATATTTGAGTATGTCAAGCGGAATTTTCCCACAACCCTTTTTTACCGAATCGCCTTAAAAAACAAAGTACAGTCAT : 2405
MarF      : ------------------------------------------------------------------------------------------- :    -
MarR      : ------------------------------------------------------------------------------------------- :    -
Mar2F     : ------------------------------------------------------------------------------------------- :    -
Mar2R     : ------------------------------------------------------------------------------------------- :    -

            460         *      2480         *      2500         *      2520         *      2540
Dwilli94  : ------------------------------------------------------------------------------------------- :    -
Dwilli95  : ------------------------------------------------------------------------------------------- :    -
Dwilli96  : ------------------------------------------------------------------------------------------- :    -
Mar       : GAGTCTTGGATTCCTTTTACCTTAATGTCATAAAAATGAATGCATATTTTGTATCGGCTATTATATGTATATATTTTTCATACTATACATA :  283
Dwilli72  : GAGTCTTGGGTTCCTTTTACCTAAATGTCATAAAAATGAATGCATATTTTGTATCGGCTATTATAT----ATATTTTTCATACTATACATA : 1987
Dtropi16  : GAGTCTTGGGTTCCTTTTACCTAAATGTCATAAAAATTAATACATATTTTGTATCGGCTTTTATAT----ATATTTTTCATACTATACATA : 2332
Dtropi29  : GAGTCTTGGGTTCCTTTTACCTAAATGTCATAAAAATTAATACATATTTTGTATCGGCTTTTATAT----ATATTTTTCATACTATACATA : 2326
Dtropi10  : GAGTCTTGGGTTCCTTTTACCTAAATGTCATAAAAATTAATACATATTTTGTATCGGCTTATATAT----ATATTTTTCATACTATACATA : 2325
Dtropi17  : GAGTCTTGGGTTCCTTTTACCTAAATGTCATAGAAATTAATACATATTTTGTATCGGCTTTTATAT----ATATTTTTCATACTATACATA : 2271
Dtropi8   : GAGTCTTGGGTTCCTTTTACCTAAATGTCATAAAAATTAATACATATTTTGTATCGGCTTTTATAT----ATATTTTTCATACTATACATA : 2330
Dtropi7   : GAGTCTTGGGTTCCTTTTACCTAAATGTCATAAAAATTAATACATATTTTGTATCGGCTTTTATAT----ATATTTTTCATACTATACATA : 2325
consensus : GAGTCTTGGGTTCCTTTTACCTAAATGTCATAAAAATTAATACATATTTTGTATCGGCTTTTATAT----ATATTTTTCATACTATACATA : 2492
MarF      : ------------------------------------------------------------------------------------------- :    -
MarR      : ------------------------------------------------------------------------------------------- :    -
Mar2F     : ------------------------------------------------------------------------------------------- :    -
Mar2R     : ------------------------------------------------------------------------------------------- :    -
```

```
                 *       2560          *       2580          *       2600          *       2620          *       2640
Dwilli94  : ------------------------------------------------------------------------------------------- :    -
Dwilli95  : ------------------------------------------------------------------------------------------- :    -
Dwilli96  : ------------------------------------------------------------------------------------------- :    -
Mar       : TATGTGCATACATGTATATCTTACATTTATATTACATTATGCATGTTCGCTTATATTATAATATACTTTTTACCCTATAGGGACAAAATAT :  374
Dwilli72  : TATGTGCATACATGTATATCTTACATTTATATTACATTATGCATGTTCGCTTATATTATAATATACTTTTTACCCTATAGGGACAAAATAT : 2078
Dtropi16  : TATGTGCACACAT-----------------ATTACATTATGCATGTTCGCTTATATTATAATATACTTTTTACCCTATAGGGACAAAATAT : 2406
Dtropi29  : TATGTGCACACAT-----------------ATTACATTATGCATGTTCGCTTATATTATAATATACTTTTTACCCTATAGGGACAAAATAT : 2400
Dtropi10  : TATGTGCACACAT-----------------ATTACATTATGCATGTTCGCTTATATTATAATATACTTTTTACCCTATAGGGACAAAATAT : 2399
Dtropi17  : TATGTGCACACAT-----------------ATTACATTATGCATGTTCGCTTATATTATAATATACTTTTTACCCTATAGGGACAAAATAT : 2345
Dtropi8   : TATGTGCACACAT-----------------ATTACATTATGCATGTTCGCTTATATTATAATATACTTTTTACCCTATAGGGACAAAATAT : 2404
Dtropi7   : TATGTGCACACAT-----------------ATTACATTATGCATGTTCGCTTATATTATAATATACTTTTTACCCTATAGGGACAAAATAT : 2399
consensus : TATGTGCACACAT-----------------ATTACATTATGCATGTTCGCTTATATTATAATATACTTTTTACCCTATAGGGACAAAATAT : 2566
MarF      : ------------------------------------------------------------------------------------------- :    -
MarR      : ------------------------------------------------------------------------------------------- :    -
Mar2F     : ------------------------------------------------------------------------------------------- :    -
Mar2R     : ------------------------------------------------------------------------------------------- :    -

            0          *       2660          *       2680          *       2700          *       2720          *
Dwilli94  : ------------------------------------------------------------------------------------------- :    -
Dwilli95  : ------------------------------------------------------------------------------------------- :    -
Dwilli96  : ------------------------------------------------------------------------------------------- :    -
Mar       : AAATAATAGCAAAATATTTTTAAAGTTGCCATGTACGAAAGTGAAGCATTAATTTTTCTGCGTGTAGCTGCGTAACAAAGCGACATAGTAC :  465
Dwilli72  : AAATAATGGCAAAATATTTTTAAAGTTGCCATGTAAGAAAGTGAAGCATTAATTTTTCTGCGTGTAGCTGCGTAACAAAGCGATATAGTAC : 2169
Dtropi16  : AAATAATGGCAAAATATTTTTAAAGTTGCCATGTAAGAAAGTGAAGCATTAATTTTTCTGCGTGTAGCTGCGTAACAAAGCGA-------- : 2489
Dtropi29  : AAATAATGGCAAAATATTTTTGAAGTTGCCATGTAAGAAAGTGAAGCATTAATTTTTCTGCGTGTAGCTGCGTAACAAAGCGA-------- : 2483
Dtropi10  : AAATAATGGCAAAATATTTTTAAAGTTGCCATGTAAGAAAGTGAAGCATTAATTTTTCTGCGTGTAGCTGCGTAACAAAGCGA-------- : 2482
Dtropi17  : AAATAATGGCAAAATATTTTTAAAGTTGCCATGTAAGAAAGTGAAGCATTAATTTTTCTGCGTGTAGCTGCGTAACAAAGCGA-------- : 2428
Dtropi8   : AAATAATGGCAAAATATTTTTAAAGTTGCCATGTAAGAAAGTGAAGCATTAATTTTTCTGCGTGTAGCTGCGTAACAAAGCGA-------- : 2487
Dtropi7   : AAATAATGGCAAAATATTTTTAAAGTTGCCATGTAAGAAAGTGAAGCATTAATTTTTCTGCGTGTAGCTGCGTAACAAAGCGA-------- : 2482
consensus : AAATAATGGCAAAATATTTTTAAAGTTGCCATGTAAGAAAGTGAAGCATTAATTTTTCTGCGTGTAGCTGCGTAACAAAGCGATATAGTAC : 2657
MarF      : ------------------------------------------------------------------------------------------- :    -
MarR      : ------------------------------------------------------------------------------------------- :    -
Mar2F     : ------------------------------------------------------------------------------------------- :    -
Mar2R     : ------------------------------------------------------------------------------------------- :    -
```

```
                2740           *    2760           *    2780           *    2800           *    2820
Dwilli94  : ------------------------------------------------------------------------------------------- :    -
Dwilli95  : ------------------------------------------------------------------------------------------- :    -
Dwilli96  : ------------------------------------------------------------------------------------------- :    -
Mar       : TGCTTTCGTCAAAAGTCTGTGCGACACTAGCTGTTTTTTTACGCTCTCAACTTGTACTTCGTGCTCACATCGGCATGCACACAGACGGCCA :  556
Dwilli72  : TGCTTTCGTCAAAAGTCTGTGCGACACTAGCTGTTTTTTTACGCTCTCAACTTGTACTTCGTGCTCACATCGGCATGCACACAGACGGCCA : 2260
Dtropi16  : ------------------------------------------------------------------------------------------- :    -
Dtropi29  : ------------------------------------------------------------------------------------------- :    -
Dtropi10  : ------------------------------------------------------------------------------------------- :    -
Dtropi17  : ------------------------------------------------------------------------------------------- :    -
Dtropi8   : ------------------------------------------------------------------------------------------- :    -
Dtropi7   : ------------------------------------------------------------------------------------------- :    -
consensus : TGCTTTCGTCAAAAGTCTGTGCGACACTAGCTGTTTTTTTACGCTCTCAACTTGTACTTCGTGCTCACATCGGCATGCACACAGACGGCCA : 2748
MarF      : ------------------------------------------------------------------------------------------- :    -
MarR      : ----------------------------------------------------TGTACTTCGTGCTCACATCG------------------- :   20
Mar2F     : ------------------------------------------------------------------------------------------- :    -
Mar2R     : ------------------------------------------------------------------------------------------- :    -

                    *     2840          *     2860          *
Dwilli94  : ------------------------------------------------------ :    -
Dwilli95  : ------------------------------------------------------ :    -
Dwilli96  : ------------------------------------------------------ :    -
Mar       : CACGAGCAATTGCCGAGCAAAAGTGCCCGCACGGGCTATGGGCGCCTACCTCTG :  610
Dwilli72  : CACGAGCAATTGCCGGACAAAAGTGCCCGCACGGGCTATGGGCGCCTACCTCTG : 2314
Dtropi16  : ------------------------------------------------------ :    -
Dtropi29  : ------------------------------------------------------ :    -
Dtropi10  : ------------------------------------------------------ :    -
Dtropi17  : ------------------------------------------------------ :    -
Dtropi8   : ------------------------------------------------------ :    -
Dtropi7   : ------------------------------------------------------ :    -
consensus : CACGAGCAATTGCCGGGCAAAAGTGCCCGCACGGGCTATGGGCGCCTACCTCTG : 2802
MarF      : ------------------------------------------------------ :    -
MarR      : ------------------------------------------------------ :    -
Mar2F     : ------------------------------------------------------ :    -
Mar2R     : ------------------------------------------------------ :    -
```
