# Supplementary material for: Mar, a MITE family of hAT transposons in Drosophila
Source: Mob DNA. 2012 Aug 31;3:13. doi: 10.1186/1759-8753-3-13 (PMC3517528; doi:10.1186/1759-8753-3-13)
Supplement: Additional file 6 — Alignment view of the putative Mar transposase and the general transcription factor II-I repeat domain-containing protein 2-like from Xenopus tropicalis. [file 1759-8753-3-13-S6.pdf]

Additional file 6

```
                      *        20         *        40         *        60         *        80         *       100         *       120
MarTransp  : -------------------------MWNVNWEKQFFFMLNISNKPECIVCNRVITKISKYCIQRHFVSIHKELHNKNEDE-KLEIFQKLNSLFNSKISRFRNFTPSHAGHSVDENVILKASF :  96
XP_0029410 : MSGMFETKSFTKNCAKKRKIDSENREFQEKWEEEYFFGKN-KDKPQCLVCAQTISSCKEYNVKRHYTTLHGAKYAKYTGEARLTIIKDLKS----KLCKQKNI---FSAVTTTQKAAVKASF : 114
                                       5   WE 25FF  N  1KP2C6VC   I3    Y 6 RH5 36H     K   E 4L I   L S    K6 4 4N        3  2   6KASF

                    *       140         *       160         *       180         *       200         *       220         *       240
MarTransp  : AVAIALARKGRPFKDGMFFKDLCLEILDILGQKEKESYSLIKDIPLSNRTITRRTEEVGQYLYSQTNQRIEGCAFFSVCLDESVDIRNISQMIICVRTIDYEFNICDEFLKLHSFHGHVNGK : 218
XP_0029410 : LVAEEIAKRKYPFSSGTLVKTCALKMAEAFGNETMA--KTVESISLSHQTVARHICTINQHLTDKLQNILRSAPYFSIALDESTDITDTCQLVIFVRSVDQEFHISEELLEIVPLHGSTKGE : 234
              VA  6A44  PF  G   K   L 6    G         6  I LS  T6 R    6 Q L       6    5FS6 LDES DI 1  Q66I VR36D EF I  E L 6   HG   G

                  *       260         *       280         *       300         *       320         *       340         *       360
MarTransp  : STFDAFEKHVLSLQLTDKISSICTDGAATMKGNNEGFLGHANRKIGEIPFFHCFIHQQALFAKMFQVVDAMGVATKIINRIKGGHNSLTHRKFKSFLVEIGAEYKDLSLFTDVRWLSRGQSL : 340
XP_0029410 : DIYNAVKATVTKYGGFSSCTCIVTDGARAMVGRNQGLAGRLRKEGIDCHMFHCIVHQEVLCGTSLKMADIMDVVTKVTNLIRGGNRSLTHRRFKNFLEELDAAYGDLLLHTNVRWLSAGKCL : 356
               51A    V         3 I TDGA  M G N2G  G   4       FHC 6HQ2 L      6 D M V TK6 N I4GG  SLTHR4FK FL E6 A Y DL L T1VRWLS G  L

                *       380         *       400         *       420         *       440         *       460         *       480
MarTransp  : DRLYSLRKEVISFLEKENLEKDKELLTSLKNPLFILDLAFLTDLVLFINQLNLQLQGRNKLPSDLIAAIKNFTRKISSLQIQMKNNDLSSLQRTAESVTLYPNPSRIQEYAEAV---NTLSS : 459
XP_0029410 : VRFFALRKEIHLYL--SDIKCDSYLLEHLTDVSFLTALAFLTDITQFLNSLNLNLQGRDQNVSQLYSHIAAFRNKLHLLKSFLAKNDLTHFESCCELFQEMKGNGVILNFSQFVPKMDILIH : 476
              R 5 LRKE6  5L   16  D  LL  L 1  F6  LAFLTD6  F6N LNL LQGR1   S L   I  F  K6  L  6   NDL3  2   E          I  5 2 V   1 L

              *       500         *       520         *       540         *       560         *       580         *       600         *
MarTransp  : EIAEKFSDFKQINWLIAVFNSPLEAESELIPEEIKKEIQVMKTDLMLPLSS--GIEFWRNIPMDKFPNSKKAMLKLSSMFTTTYICESTFSTMKLIKDSIRNSLTDSHLEDLLRIKSYPNNI : 579
XP_0029410 : EFNTRFQDFEGMEASFKLFNNPINTDIAAQDTKYHMELCELQGDQFLHSCSEKGVAFFKLLSQEQYPILRDFGMRMASMFGSTYICEKAFSDMGFIKSKYRNSICDSTLQQIIRISTTSISA : 598
             E   4F DF  6     6FN P6             E6  6  D  L   S  G6 F54 6    5P  4   646 SMF 3TYICE  FS M  IK   RNS6 DS L2 66RI 3

                    620
MarTransp  : EVKETV-EFLNKN--- : 591
XP_0029410 : DIDELVAQQLHPQTSH : 614
              6 E V 2 L
```

Alignment view of the putative *Mar* Transposase and the general transcription factor II-I repeat domain-containing protein 2-like from *Xenopus tropicalis*. The box in c-terminal region represent the *hAT* dimerization domain.
