# Supplementary material for: Mar, a MITE family of hAT transposons in Drosophila
Source: Mob DNA. 2012 Aug 31;3:13. doi: 10.1186/1759-8753-3-13 (PMC3517528; doi:10.1186/1759-8753-3-13)
Supplement: Additional file 8 — Comparison of the TSD consensus sequences and TIRs from Mar and five different D. willistoni hAT elements. [file 1759-8753-3-13-S8.pdf]

Additional file 8

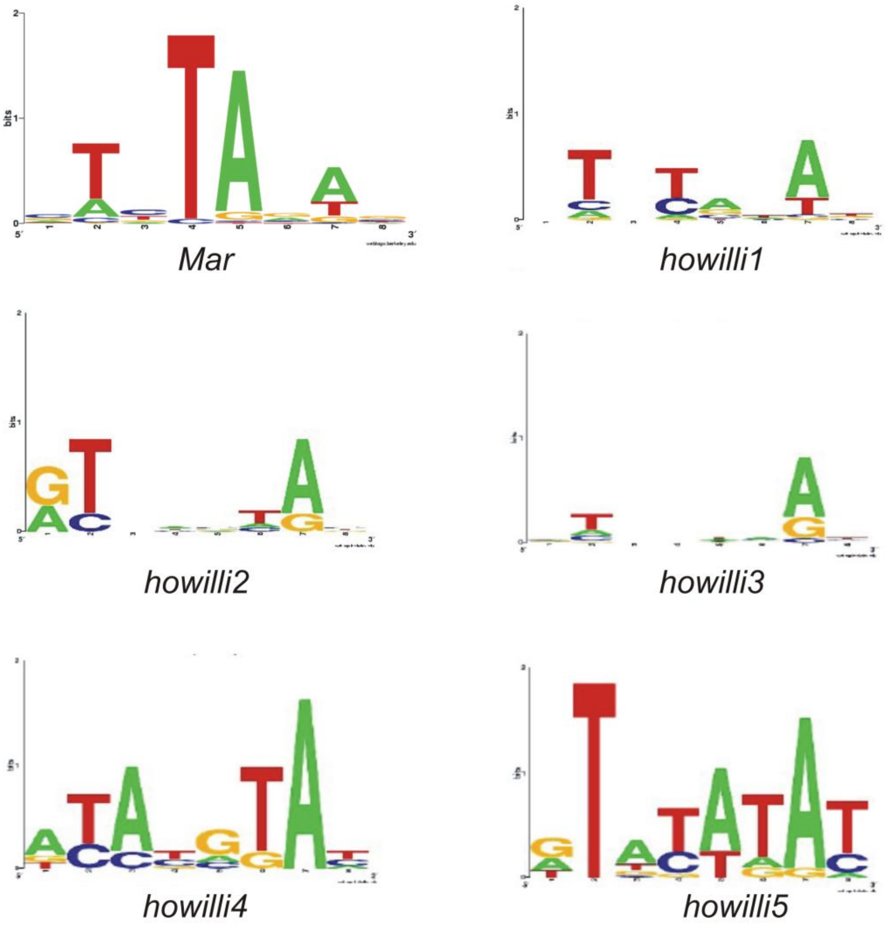

Sequence logo analysis for the 8 bp TSDs observed for *Mar* and for the *hAT* elements found in *D. willistoni* (Ortiz *et al*. 2010, Genetica 138:649-655). The y-axis shows 2 bits of information and the x-axis represents the nucleotide position in the TSDs.

```
Howilli4 : TAGAGAGCTGC-- : 11
Howilli5 : TAGACAGCTGC-- : 11
Howilli2 : CAGAGAACTGCAA : 13
Mar      : CAGRGGTAGGC-- : 11
Howilli1 : TAGTGTTGGGT-- : 11
Howilli3 : TAGTGATGTAAA- : 12
```

TIR sequences comparison from *Mar* and other *hAT* elements from *D. willistoni*.
