# Supplementary material for: Mar, a MITE family of hAT transposons in Drosophila
Source: Mob DNA. 2012 Aug 31;3:13. doi: 10.1186/1759-8753-3-13 (PMC3517528; doi:10.1186/1759-8753-3-13)
Supplement: Additional file 9 — Identity and accession number of the sequences used to infer the hAT transposase phylogeny. [file 1759-8753-3-13-S9.pdf]

Additional file 9

Identity and accession number of the sequences used to infer the *hAT* transposase phylogeny

Name	Accession number	Species	Database/manuscript
AeBuster1	ABF20543	*Aedes aegypti*	GENBANK
AeBuster2	ABF20544	*Aedes aegypti*	GENBANK
AmBuster1	EFB22616	*Ailuropoda melanoleuca*	GENBANK
BtBuster1	ABF22695	*Bos taurus*	GENBANK
CfBuster1	ABF22696	*Canis lupus familiaris*	GENBANK
CsBuster	ABF20548	*Ciona savignyi*	GENBANK
Daysleeper	CAB68118	*Arabidopsis thaliana*	GENBANK
DrBuster2	ABF20550	*Danio rerio*	GENBANK
DrBuster1	ABF20549	*Danio rerio*	GENBANK
EcBuster1	XP_001504971	*Equus caballus*	GENBANK
Hermes	AAC37217	*Musca domestica*	GENBANK
Hermit	U22467	*Lucilia cuprina*	GENBANK
Herves	AAS21248	*Anopheles gambiae*	GENBANK
Hobo	A39652	*Drosophila melanogaster*	GENBANK
Homer	AAD03082	*Bactrocera tryoni*	GENBANK
Hopper-we	AAL93203	*Bactrocera dorsalis*	GENBANK
HsBuster1	AAF18454	*Homo sapiens*	GENBANK
IpTip100	BAA36225	*Ipomoea purpurea*	GENBANK
MamBuster2	XP_001108973	*Macaca mulatta*	GENBANK
MmBuster2	AAF18453	*Mus musculus*	GENBANK
PtBuster2	ABF22699	*Pan troglodytes*	GENBANK
Restless	CAA93759	*Tolypocladium inflatum*	GENBANK
RnBuster2	NP_001102151	*Rattus norvegicus*	GENBANK
SpBuster1	ABF20546	*Strongylocentrotus purpuratus*	GENBANK
SpBuster2	ABF20547	*Strongylocentrotus purpuratus*	GENBANK
SpBuster2_b	NM_001114196	*Strongylocentrotus purpuratus*	GENBANK
SpBuster2_c	DQ481199	*Strongylocentrotus purpuratus*	GENBANK
TcBuster	ABF20545	*Tribolium castaneum*	GENBANK
Tol2	BAA87039	*Oryzias latipes*	GENBANK
Tramp	CAA76545	*Homo sapiens*	GENBANK
xenopus_factorII	XM_002941008	*Xenopus tropicalis*	GENBANK
XtBuster	ABF20551	*Xenopus tropicalis*	GENBANK
AeBuster3	TF001186	*Aedes aegypti*	TEFam
AeBuster4	TF001187	*Aedes aegypti*	TEFam
AeBuster5	TF001188	*Aedes aegypti*	TEFam
AeBuster7	TF001336	*Aedes aegypti*	TEFam
AeHermes2	TF0013338	*Aedes aegypti*	TEFam
AeTip100_2	TF000910	*Aedes aegypti*	TEFam
CxKink2	TF001637	*Culex pipiens quinquefasciatus*	TEFam
Ac-like1 (hAT-6_DR)		*Danio rerio*	REPBASE
Ac-like2 (hAT-7_DR)		*Danio rerio*	REPBASE
hAT1_DP (hAT-1_DPp)		*Drosophila pseudoobscura*	REPBASE
hAT2_AG (hAT-2_AG)		*Anopheles gambiae*	REPBASE
hAT-5_DR		*Danio rerio*	REPBASE
MlBuster1 (hAT-4_ML)		*Myotis lucifugus*	REPBASE
Myotis_hAT1 (Myotis_hAT1-1p)		*Myotis lucifugus*	REPBASE
sm_hat6 (hAT-6_SM_1p)		*Schmidtea mediterranea*	REPBASE
sm_hat3 (hAT-3_SM_1p)		*Schmidtea mediterranea*	REPBASE
Hoana1		*Drosophila ananassae*	Ortiz and Loreto (2009)
Hoana3		*Drosophila ananassae*	Ortiz and Loreto (2009)

Hoana6	*Drosophila ananassae*	Ortiz and Loreto (2009)
Hoana7	*Drosophila ananassae*	Ortiz and Loreto (2009)
Hoana8	*Drosophila ananassae*	Ortiz and Loreto (2009)
Homo1	*Drosophila mojavensis*	Ortiz and Loreto (2009)
Homo3	*Drosophila mojavensis*	Ortiz and Loreto (2009)
Homo5	*Drosophila mojavensis*	Ortiz and Loreto (2009)
Homo7	*Drosophila mojavensis*	Ortiz and Loreto (2009)
Homo8	*Drosophila mojavensis*	Ortiz and Loreto (2009)
Hosec1	*Drosophila sechellia*	Ortiz and Loreto (2009)
Hosim1	*Drosophila simulans*	Ortiz and Loreto (2009)
Howilli1	*Drosophila willistoni*	Ortiz and Loreto (2009)
Howilli2	*Drosophila willistoni*	Ortiz and Loreto (2009)
Howilli3	*Drosophila willistoni*	Ortiz and Loreto (2009)
